# A multi-targeting bionanomatrix coating to reduce capsular contracture development on silicone implants

**DOI:** 10.1186/s40824-023-00378-7

**Published:** 2023-04-22

**Authors:** Patrick Hwang, Chung Min Shin, Jennifer A. Sherwood, DongHo Kim, Vineeth M. Vijayan, Krishna C. Josyula, Reid C. Millican, Donald Ho, Brigitta C. Brott, Vinoy Thomas, Chul Hee Choi, Sang-Ha Oh, Dong Woon Kim, Ho-Wook Jun

**Affiliations:** 1 Endomimetics, LLC, Birmingham, AL 35242 USA; 2 Department of Biomedical Engineering, University of Alabama at Birmingham, 806 Shelby, 1825 University Boulevard, Birmingham, AL 35294 USA; 3 Department of Plastic and Reconstructive Surgery, Chungnam National University College of Medicine, Daejeon, 35015 Republic of Korea; 4 Department of Microbiology, Chungnam National University College of Medicine, Daejeon, 35015 Republic of Korea; 5 Department of Biomedical Engineering, Alabama State University, Montgomery, AL 36104 USA; 6 Department of Pediatric Dentistry, University of Alabama at Birmingham, Birmingham, AL 35294 USA; 7 Department of Medicine and Division of Cardiovascular Disease, University of Alabama at Birmingham, Birmingham, AL 35233 USA; 8 Department of Material Science and Engineering, University of Alabama at Birmingham, Birmingham, AL 35294 USA; 9 Department of Anatomy and Cell Biology, Brain Research Institute, College of Medicine, Chungnam National University College of Medicine, Daejeon, 35015 Republic of Korea

**Keywords:** Capsular contracture, Fibrotic tissue formation, Myofibroblast differentiation, Inflammation, Nitric oxide, Bionanomatrix

## Abstract

**Background:**

Capsular contracture is a critical complication of silicone implantation caused by fibrotic tissue formation from excessive foreign body responses. Various approaches have been applied, but targeting the mechanisms of capsule formation has not been completely solved. Myofibroblast differentiation through the transforming growth factor beta (TGF-β)/p-SMADs signaling is one of the key factors for capsular contracture development. In addition, biofilm formation on implants may result chronic inflammation promoting capsular fibrosis formation with subsequent contraction. To date, there have been no approaches targeting multi-facted mechanisms of capsular contracture development.

**Methods:**

In this study, we developed a multi-targeting nitric oxide (NO) releasing bionanomatrix coating to reduce capsular contracture formation by targeting myofibroblast differentiation, inflammatory responses, and infections. First, we characterized the bionanomatrix coating on silicon implants by conducting rheology test, scanning electron microcsopy analysis, nanoindentation analysis, and NO release kinetics evaluation. In addition, differentiated monocyte adhesion and *S. epidermidis* biofilm formation on bionanomatrix coated silicone implants were evaluated in vitro. Bionanomatrix coated silicone and uncoated silicone groups were subcutaneously implanted into a mouse model for evaluation of capsular contracture development for a month. Fibrosis formation, capsule thickness, TGF-β/SMAD 2/3 signaling cascade, NO production, and inflammatory cytokine production were evaluated using histology, immunofluorescent imaging analysis, and gene and protein expression assays.

**Results:**

The bionanomatrix coating maintained a uniform and smooth surface on the silicone even after mechanical stress conditions. In addition, the bionanomatrix coating showed sustained NO release for at least one month and reduction of differentiated monocyte adhesion and *S. epidermidis* biofilm formation on the silicone implants in vitro. In in vivo implantation studies, the bionanomatrix coated groups demonstrated significant reduction of capsule thickness surrounding the implants. This result was due to a decrease of myofibroblast differentiation and fibrous extracellular matrix production through inhibition of the TGF-β/p-SMADs signaling. Also, the bionanomatrix coated groups reduced gene expression of M1 macrophage markers and promoted M2 macrophage markers which indicated the bionanomatrix could reduce inflammation but promote healing process.

**Conclusions:**

In conclusion, the bionanomatrix coating significantly reduced capsular contracture formation and promoted healing process on silicone implants by reducing myfibroblast differentiation, fibrotic tissue formation, and inflammation.

**Graphical Abstract:**

A multi-targeting nitric oxide releasing bionanomatrix coating for silicone implant can reduce capsular contracture and improve healing process. The bionanomatrix coating reduces capsule thickness, α-smooth muscle actin and collagen synthesis, and myofibroblast differentiation through inhibition of TGF-β/SMADs signaling cascades in the subcutaneous mouse models for a month.

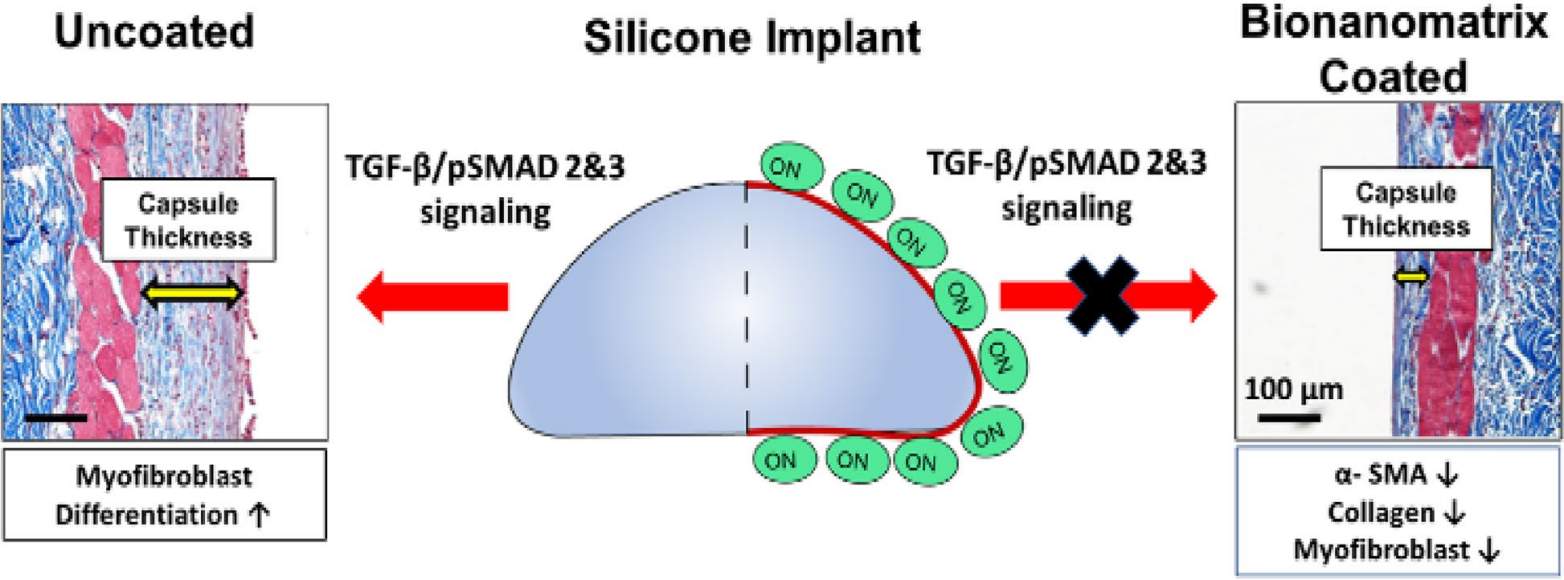

**Supplementary Information:**

The online version contains supplementary material available at 10.1186/s40824-023-00378-7.

## Background

Reconstructive surgeries are commonly conducted using silicone implants for breast reconstruction and are also used in various other locations including the face and hip. A major complication of reconstruction surgeries is capsular contracture caused by the formation of fibrotic tissue [1]. Capsular contracture can be painful and typically results in the implanted areas becoming asymmetrically displaced or otherwise deformed [1]. Capsular contracture results from an excessive fibrotic foreign body reaction which may occur after implantation [2, 3]. Clinical risk factors for capsular contracture include surgical position of the implant, site of the implant incision, skin or biofilm infection, hematoma formation post-operatively, and the tendency for development of hypertrophic scar formation [4, 5]. Subclinical infection resulting from the implantation procedure and hematoma formation, with biofilm formation, plays an important role in contracture development [6–9]. In fact, the removal of implants that have induced severe capsular contracture demonstrate the presence of biofilms. Recent studies have reported that biofilms on implants may also promote chronic inflammation leading to the formation of severe capsular fibrosis with subsequent contraction [10, 11]. All the above clinical risk factors may promote the development of capsular contracture by stimulating inflammatory responses and fibrotic tissue formation.

Fibroblasts, macrophages, and lymphocytes are reported to be the predominant cell types within capsular contracture [12–14]. Fibroblasts accumulate at the ‘contact zone’ between the implant and the capsule and produce collagen initiating formation of the capsule [1]. As the severity of capsular contracture increases, collagen fibers become thicker and resemble cable-like bundles surrounding the implant [2, 15, 16]. Transforming growth factor beta (TGF-β), mostly produced from fibroblasts, has been widely considered a ‘master switch’, which is critical for promoting capsular contracture by activating fibroblast differentiation into myofibroblasts, as well as stimulating extracellular matrix (ECM) production through its TGF-β/phosphorylated-small mothers against decapentaplegics (p-SMADs) signaling cascades [17–19]. Myofibroblasts are contractile fibroblasts which provide contractile force and have been found on the outer layer of capsules constituting, on average, 27% of the capsular thickness. Thus, myofibroblasts may play a key role in the development of capsular contracture [20, 21]. In addition, macrophages and mast cells have been investigated for their involvement in the process of capsular contracture formation by promoting foreign body reaction through the production of pro-inflammatory and pro-fibrotic cytokines, such as tumor necrosis factor alpha (TNF-α), histamine, and TFG-β [13, 15].

Once capsular contracture forms, the gold standard treatment is complete resection of the fibrotic tissue and implant, resulting in additional risks to the patient including increased chance of infection, scar tissue formation and recurrence of capsular contracture, as well as prolonged recovery time [1, 22, 23]. Therefore, alternative preventative strategies are needed to reduce the risk of capsular contracture development and, as a result, the need for future surgical intervention. Medical treatment can also be considered to prevent capsular contracture formation including leukotriene inhibitors, antibiotics, or botulinum toxin which target reduced inflammation, infection, or fibrotic tissue formation, respectively [24–28]. However, these medications may require repeated treatments and have unwanted side effects, such as hepatic dysfunction and flu-like symptoms [24–26]. There have been several alternative preventative strategies that have been developed to combat capsular contracture development. One such strategy that has been shown to reduce capsular contracture is topographical modification [29, 30]. Recently, textured implants such as macrotexture and nanotexture type implants have been developed to overcome the shortcomings of the widely used smooth type surface implants. However, concerns have been raised about both topographical modifications; nanotexture surface implants shift from the implant location whereas macrotexture implants promote frequent inflammatory responses [30]. Moreover, it has been reported by the US Food and Drug Administration (FDA) that textured surface implants increase the risk of breast implant-associated lymphoma (BIA-ALCL) [31, 32].

Additionally, various coating strategies have been explored to reduce contracture development. Polyurethane-coated implants have been shown to reduce the risk of contracture development; however, the degradation byproducts could be toxic [33]. Furthermore, the coating can produce future complications such as capsular contracture and hematoma [33, 34]. Anti-bacterial coatings have been developed to reduce chronic inflammation at the implantation site. Several studies have been done on the efficacy of plasma activation of the implant surface to reduce biofilm formation [35, 36]. Although it has been shown to reduce capsular contracture, the coating has been shown to be only effective against early bacterial infection, leaving the site susceptible to delayed infection or capsular contracture [36]. Various anti-adhesive coatings have been explored to avoid foreign body responses [37–39]. Zwitterionic polydopamine coatings exhibit anti-fouling properties by forming a hydration layer on implants [37]. Additionally, antiadhesive barrier solutions (AABS) such as Guardix-SG and hyaluronic acid applied on implants have been shown to reduce inflammation [38, 39]. To date, there have been no approaches designed to target the multi-faceted mechanisms that lead to capsular contracture including myofibroblast differentiation, fibrotic tissue formation, inflammation, and biofilm formation.

**Scheme 1:**
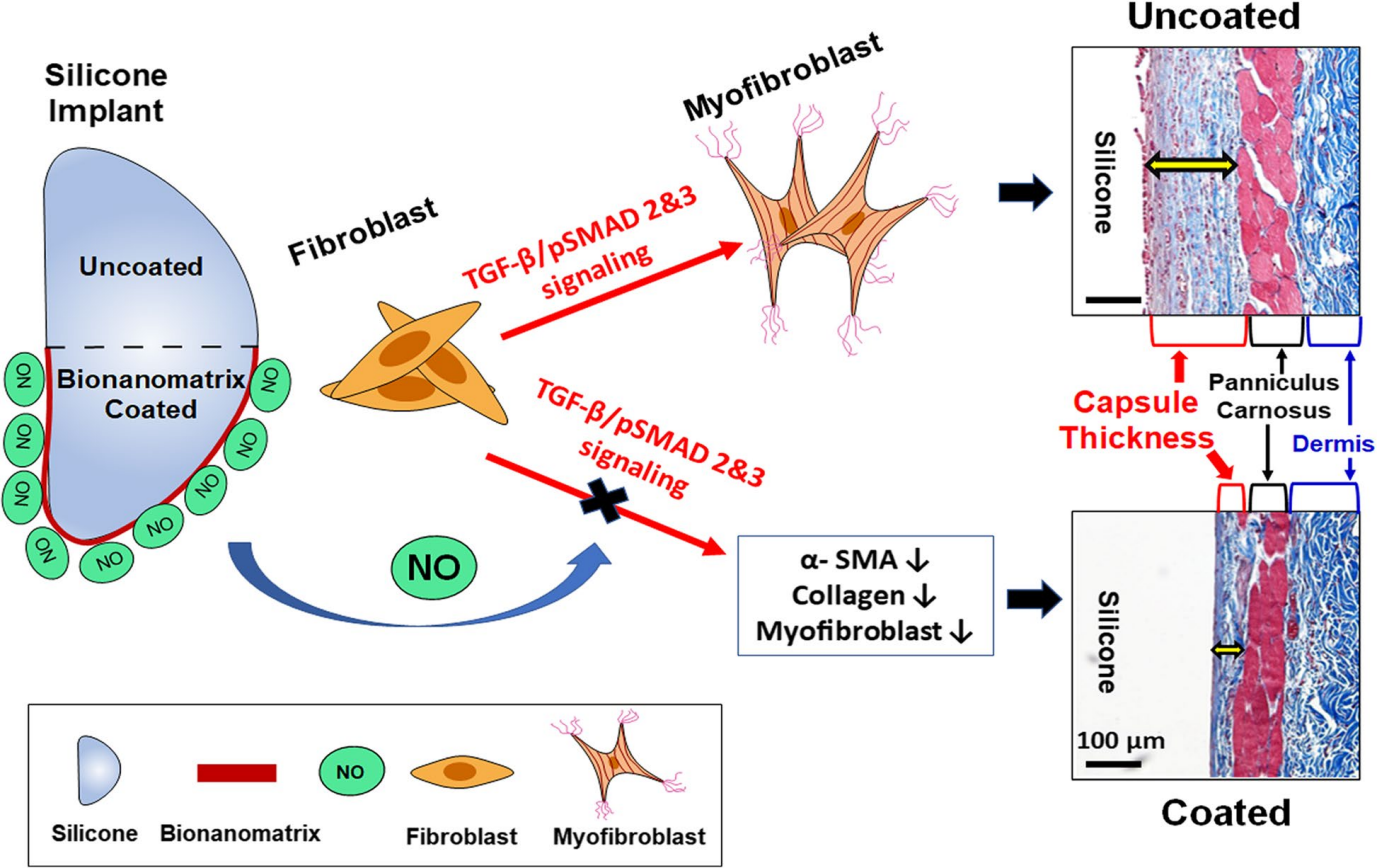
Application of bionanomatrix coating to reduce capsular contracture formation on either bionanomatrix coated or uncoated silicone implants

In this study, the bionanomatrix coating was applied to the silicone implants targeting multiple mechanisms of capsular contracture formation to overcome the limitations of other approaches (Scheme 1). Our bionanomatrix coating provides sustained NO release, which reduces myofibroblast differentiation by inhibition of TGF-β signaling, inflammatory responses, infection, and fibrotic tissue formation [40–43]. The highly biocompatible bionanomatrix is composed of two peptide amphiphiles (PAs) which comprise hydrophobic alkyl tail (palmitic acid) attached to the hydrophilic bioactive functional peptides: PA-YIGSR [CH_3_(CH2)_14_CONH-GTAGLIGQ-YIGSR] and PA-KKKKK [CH_3_(CH_2_)_14_CONH-GTAGLIGQ-KKKKK] [40, 42]. This amphiphilic property allows self-assembly of PAs into cylindrical micelle nanofibers and these nanofibers form bionanomatrix [44, 45]. The PAs contain an enzyme-mediated degradation sequence (GTAGLIGQ) connected to either a cell adhesive ligand (YIGSR) or a NO producing donor sequence (KKKKK) [40–42]. The self-assembled bionanomatrix can be coated onto silicone implants using an ultrasonic spray coating approach, which avoids using toxic solvents thereby minimizing the risk of inflammation [42]. NO release kinetics from the bionanomatrix coating are controlled by the functional groups (GTAGLIGQ) and the structure of bionanomatrix. NO is released by dissociation from the surface of the coating, and then gradual biodegradation of the bionanomatrix, which exposes layers of the bionanomatrix [40, 42, 46]. Our previous results demonstrated that the bionanomatrix provided sustained release of NO at least over a month period and did not produce any foreign body response or negative effects on cells or tissues [40, 42, 46]. NO has been shown to inhibit bacterial infection or biofilm formation, reduce inflammatory responses, and importantly NO prevents myofibroblast differentiation and fibrotic tissue formation through inhibiting TGF-β/p-SMADs signaling [43, 47–51]. Thus, we hypothesized that the bionanomatrix coating for silicone implants will provide sustained NO release and prevent the capsular contracture formation by targeting the key mechanisms described above.

First, we characterized the NO releasing bionanomatrix coating on silicone implants in vitro. Then, we subcutaneously implanted the bionanomatrix coated silicone implants into mice for 30 days to evaluate capsular contracture in vivo. Here, our multi-targeting bionanomatrix demonstrates a great potential to overcome current limitations for prevention of capsular contracture by reducing unwanted side effects and the need for additional reconstructive surgery. These outcomes can be applied to various medical devices and implants including prosthetic implants, cardiac implantable electronic devices, grafts, and mechanical circulatory devices.

## Methods

### Bionanomatrix synthesis

F-moc chemistry was used to synthesize peptides using an Apex 396 peptide synthesizer using solid-peptide synthesis method with successive addition of protected amino acids to a growing peptide chain immobilized on a solid phase; which was previously described to synthesize the peptide sequence GTAGLIGQ-YIGSR and GTAGLIGQ-KKKKK [40, 46, 52, 53]. The synthesized peptide sequences were alkylated with palmitic acid (CH_3_(CH_2_)_14_COOH) to create the peptide amphiphiles (PAs) using a manual coupling reaction. This reaction was conducted in a solution mixture consisting of hexafluorophosphate (HBTU), N,N-diisopropylethylamine (DIEA), and dimethylformamide (DMF) for 24 h at room temperature [54]. The resulting alkylated PAs were then cleaved from the resin in a solution containing trifluoroacetic acid (TFA), triisopropylsilane (TIPS), deionized water, and anisole (M_R_ 40:1:1:1) for 2 h at room temperature; the excess TFA was then removed via rotary evaporation [54]. The resulting process created PA-YIGSR and PA-KKKKK. PA-YIGSR and PA-KKKKK (9:1 ratio) was reacted with NO gas passed through 5 M potassium hydroxide (to remove impurities such as higher oxide species) under Argon gas atmosphere overnight at room temperature to create PA-YK-NO [40, 46]. The PA-YK-NO layered deposition during coating application to the silicone surface using an ultrasonic spray coating machine (Exactacoat, Sonotek Corporation) forms the bionanomatrix.

### Bionanomatrix coating on silicone implants

An ultrasonic spray coating machine (Exactacoat, Sonotek Corporation) was used to apply the bionanomatrix coating to the surface of silicone implants [42]. The silicone surface was coated using a wide-spray Impact nozzle in a back-and-forth pattern (flow rate: 0.75, speed: 50 mm/sec, shaping air: 1.5 PSI, drying time: 90 secs), and each layer was allowed to completely dry before application of another [42]. Silicone implants were coated with 30-, and 60-layer thicknesses for in vitro coating characterization.

### Rheology test

The contact shear stress experiment was performed on the bionanomatrix coated silicone implants using an AR-2000 rheometer to analyze coating stability (TA Instruments) [55]. The samples were placed on a parallel plate/plate configuration on the rheometer followed by the application of different stress ranges, more specifically 30–100 Pa (typical silicone implant contact shear stress after breast implantation) and 300–500 Pa (potential increase of contact shear stress based on silicone implant designs) [56, 57]. After the subjection of the shear stresses, samples were removed from the parallel plate and used for further experiments such as scanning electron microscopy to assess the stability of the coating.

### Coating characterization

#### Scanning electron microscope (SEM) analysis

A SEM (Quanta 650 FEG, FEI) was used to characterize the bionanomatrix coating on the silicone implants [42, 58]. The bionanomatrix coated samples were loaded on the stubs through contact with conductive SEM adhesive tape. The samples were loaded in the machine and imaged at an accelerating voltage of 10 kV with secondary electrons (SE) mode to examine detailed coating surface information.

#### X-ray photoelectron spectroscopy (XPS) analysis

The XPS surface analysis was carried out using a Phi 5000 Versaprobe from Phi Electronics, Inc [59]. The source of the X-ray used was a monochromatic Al K- alpha source (E = 1486.6 eV) at 25 W with a 100 μm spot size. The Mg anode (λ = 1253.6 eV) was used at 300 W and a barium oxide neutralizer eliminated charging. The survey scans (8 scans averaged per analysis) were obtained using pass energy of 187.5 eV with a step size of 0.5 eV. The high-resolution scans (16 scans average per analysis) were obtained with pass energy of 23.5 eV and a step size of 0.1 eV [59].

#### Nanoindentation analysis

Hardness and Young’s modulus of the bionanomatrix coated and uncoated control silicone implants were measured using an MTS NanoIndenter XP instrument with a Berkovich diamond tip (nominal radius of 50 nm) [59]. Tip calibration was performed on the fused silica standard (an accepted Young’s modulus of 72 GPa) before and after testing all silicone implants [59]. The measured Young’s modulus and hardness values were determined at the maximum load. An indentation depth of 1000 nm was used for all the measurements.

#### NO release kinetics

The bionanomatrix coated silicone implants were placed in 48 well plates with 1 wt. % agarose gel to mimic NO release in subcutaneous tissue [60–62]. The gels were collected at day 1 and at 3 to 4- day intervals up to 1 month. Each silicone sample was replaced on fresh gel after collection. Released NO was collected then and measured using a Total NO Kit (Thermo Fisher) [42]. The kit contains nitrate reductase, which converts nitrate to nitrite. The converted nitrite is combined with Griess reagents (sulfanilamide and N-(1-naphthyl)-ethylenediamine dihydrochloride) to create an azo compound for colorimetric analysis [55]. Absorbance was measured in a microplate reader at 540 nm.

### Differentiated monocyte adhesion Test

In a 24 well plate, U937 cells (10^5^ cells/well) were seeded on top of the bionanomatrix coated and uncoated control silicone implants. Cells were differentiated with phorbol 12-myristate 13-acetate (PMA; 20 ng/mL, Millipore Sigma) and TNF-α (20 ng/mL, Millipore Sigma) [63]. Evaluation of differentiated monocytes adhesion was measured at 48 h and 7 days by Calcein AM staining (4 μM, Thermo Fisher) under observation with a Nikon TE2000-S fluorescence microscope [42]. For viable cell image analysis, five areas were selected (center and four each corners) in each silicon sample to count adhered cells and average the counted cell numbers. Then, these values from more than four samples in each group were averaged again for comparison among groups.

In addition, DNA contents of adhered cells were quantified using CyQUANT assay (Invitrogen). After 48 h and 7 days of culture, the cell seeded silicone implant samples were frozen at – 70 °C. Then, the samples were thawed and the CyQUANT fluorescence GR dye/Cell lysis buffer was added to the samples. The fluorescence intensity was measured using the microplate reader (Ex: 480 & Em: 520 nm, BioTek).

### Biofilm formation assay

Biofilm formation on the bionanomatrix coated and uncoated control silicone implants was assessed using a biofilm formation assay previously described [37, 64]. Briefly, overnight cultures of *S. epidermidis* were added to 0.1 mL tryptic soy broth (TSB) and adjusted to an OD_600_ of 0.1 and added to a 12-well plate containing coated and uncoated silicone implants. The plates were then incubated at 37 °C for 48 h. After incubation, the silicone substrates were washed 3 times with phosphate buffered saline (PBS) and stained using crystal violet (0.1% wt/vol, Sigma-Aldrich). Silicone substrates were then washed 3 times with PBS, air-dried at room temperature and the stained biofilm was eluted with 30% acetic acid. The eluate was then quantified on a microplate reader at OD_600_ [37]. For SEM analysis of biofilm formation was performed with minor modification, as reported [65]. Following 48 h-incubation, silicone substrates were also fixed with 4% paraformaldehyde (HanLab) for 2 h. Each sample was rinsed three times with PBS. Samples were then dehydrated in ethanol − water mixtures, with increasing ethanol concentration gradients (60, 70, 80, 90, and 100%), and finally air-dried. Samples were coated using an ion sputter coater (GSEM, G10) for 40 s and imaged using a SEM (COXEM, EM-30) with the voltage set to 10 kV. The biofilm formation was analyzed on three separate occasions and five replicates for each sample were used.

#### Colony Forming Unit (CFU) on Silicone

*S. epidermidis* was cultured in TSB at 37 °C. Overnight cultures were added to 0.1 mL TSB and adjusted to an OD_600_ of 0.1, added to a 12-well plate containing either coated or uncoated silicone implants, and then placed in a non-shaking incubator for 48 h at 37 °C. Following incubation, planktonic cells were extracted from the cultures and every silicone substrate was rinsed three times with PBS. Silicones were transferred to 12-well flat-bottom microplates containing 1 ml PBS and sonicated in water bath sonicator at 40 kHz for 5 min. Bacterial cells were plated onto tryptic soy agar (TSA) with serial dilution. The number of viable colonies were counted and the CFU/ml values were calculated as follows: (No. of colonies X Total dilution factor) / Volume of culture plated in ml.

### Implantation of bionanomatrix coated silicone implants

Six-week-old male C57BL/6 mice (Samtako Bio Korea, Osan, Korea) were used for silicone implantation in accordance with the Guidelines for the Care and Use of Laboratory Animals and approved by Institutional Animal Care and Use Committee of Chungnam National University (CNUH-018-A0034). The animal protocol also followed the ethical guidelines of the National Institutes of Health and International Association for the Study of Pain. Smooth silicone implants (10 mm X 10 mm X 1 mm) coated with PA-YK-NO solution were sterilized using ethylene oxide gas, and implanted into the dorsum [37, 66]. A 1 cm incision was created, and a 1.5 cm pocket was made to insert the silicone implants. Uncoated silicone implants were used as the control group. The skin incision was closed, and the mice were monitored for 30 days. The animals were then sacrificed, and the implant and surrounding tissues were harvested for immunohistochemistry, fluorescent microscopy, and qRT-PCR.

### Histological analysis

The tissue surrounding both the uncoated and coated silicone implants was fixed using 10% neutral buffered formalin for three days and embedded in paraffin, dehydrated in a series of % alcohol solutions, and embedded in paraffin. The paraffin was then cut into 4 um thick sections, stained with hematoxylin and eosin, Masson’s trichrome staining, and visualized under a light microscope and Pannoramic MIDI II (3DHISTECH Ltd, Budapest, Hungary). Thickness of each capsule was measured as previously described and the mean thickness was calculated [37, 66].

### Immunofluorescence microscopy

Additional sections were dewaxed, rehydrated and incubated with anti-α-smooth-muscle-actin antibodies (α-SMA, 1:400, A5228; Sigma-Aldrich), p-SMAD2 (1:100, #Ab3101, Abcam), and p-SMAD3 (1:500, #Ab9520, Abcam) at 4 °C overnight. The sections were then incubated with anti-mouse IgG secondary antibody (1:400, BA-2000; Vector Labs) for 1 h at room temperature as previously described [37]. For immunofluorescent imaging, the sections were additionally incubated with Cy3-streptavidin secondary antibody (1:400, PA43001; GE Healthcare for 2 h at room temperature. Hoechst 33,342 (H3570; Invitrogen) stain was used to stain nuclei, and an Leica DM2500 microscope (Leica, Wetzlar, Germany) was used to visualize the sections.

### Reverse transcription PCR analysis of cytokine expression

Growth factor expression involved with capsule formation around the silicone implants was analyzed by RNA expression of markers for fibrosis formation (*TGF-β1, collagen type Iα, IαII, CTGF*), NO production (*eNOS, iNOS*) and inflammatory related cytokines (*TNF-α, IL-6, IL-1B, CD80, CD86, MMP12, IL-10, CD206, and CD68*). Trizol reagent was used to extract the total RNA from the capsule tissues surrounding the implant. cDNA synthesis was then performed in a 20 μl reaction mixture using TOPscript RT DryMix (Enzynomics, Daejeon, South Korea). Quantitative real-time polymerase chain reaction (qPCR) was performed in a 10 μl reaction mixture (10 pM primer, 4 μl cDNA, and 5 μl SYBR Green 2 × mixture) under the following conditions: 95 °C for 10 min and then 40 cycles at 95 °C for 15 s and 60 °C for 1 min using a CFX Real-Time PCR System (Bio-Rad, Hercules, CA, USA). The primer sequences (Cosmo Genetech, Daejeon, South Korea) used for qPCR were as follows: *TGF-β1* (NM_011577.2), forward: 5′-CCCTATATTTGGAGCCTGGA-3′, reverse: 5′-CTTGCGACCCACGTAGTAGA-3′, TNF-a (NM_013693.3), forward:5’-ATG AGA AGT TCC CAA ATG GCC T-3’, reverse: 5’-TCC ACT TGG TGG TTT GCT ACG-3’, *COL IαI* (NM_007742.4), forward:5’-GCACGAGTCACACCGGAACT-3’, reverse: 5’-AAGGGAGCCACATCGATGAT-3’, *Col IαII* (NM_007743), forward:5’-AGC TTT GTG GAT ACG CGG AC-3’, reverse: 5’-TAG GCA CGA AGT TAC TGC AAG-3’, *CTGF* (NM_010217), forward:5’-GAC CCA ACT ATG ATG CGA GCC-3’, reverse: 5’-TCC CAC AGG TCT TAG AAC AGG-3’, *eNOS* (NM_008713.4), forward:5’-ACC CAG GTT TCC TCG AGT AA-3’, reverse: 5’-GGC TCT GTA ACT TCCT TGG A-3’, iNOS (NM_001313922.1), forward: 5’-CAGAGGACCCAGAGACAAGC-3’, reverse: 5’-TGCTGAAACATTTCCTGTGC-3’, *CD86* (BC013807.1), forward: 5’-TCT CCA CGG AAA CAG CAT CT-3’, reverse: 5’-CTT ACG GAA GCA CCC ATG AT-3’, *IL-1β* (NM_008361.4), forward: 5’-TTG TGG CTG TGG AGA AGC TGT-3’, reverse: 5’-AAC GTC ACA CAC CAG CAG GTT-3’, MMP12 (NM_001320077.1), forward: 5’-TGA GGC AGG AGC TCA TGG A-3’, reverse: 5’-AGG CTT GAT TCC TGG GAA GTG T-3’, *CD206* (NM_008625.2), forward: 5’-CAG GTG TGG GCT CAG GTA GT-3’, reverse: 5’-TGT GGT GAG CTG AAA GGT GA-3’, *CD68* (NM_001291058), forward: 5’-ACT TCG GGC CAT GTT TCT CT-3’, reverse: 5’-GCT GGT AGG TTG ATT GTC GT-3’, *IL10* (NM_010548.2), forward: 5’-GCT CTT ACT GAC TGG CAT GAG-3’, reverse: 5’-CGC AGC TCT AGG AGC ATG TG-3’. The mRNA levels of each target gene were normalized to that of *18S rRNA* (*Rn18s*, NR_003278, forward: 5’-GCA ATT ATT CCC CAT GAA CG-3’, reverse: 5’-GGC CTC ACT AAA CCA TCC AA-3’). Relative expressions of the target genes were calculated using a comparative CT method, the relative transcription of the target genes are reported as the n-fold difference relative transcription of the house keeping genes (*18S rRNA*) and compared to the coated silicone group as a control sample. The fold-changes of the mRNA levels were calculated using the 2 − ΔΔCt method, as described previously [67].

### Western blot analysis

Capsular tissues were dissected and homogenized in cold RIPA buffer. Homogenized tissue samples were centrifuged at 15,000 RPM for 30 min, and the supernatant was used for quantitation with Bradford assay (Bio-Rad, Hercules, CA, USA). 35 μg of each lysate was separated on 8 or 10% SDS–polyacrylamide gels and transferred on polyvinylidene fluoride (PVDF) membranes (Millipore). The blotted membrane was incubated overnight at 4 °C with the appropriate primary antibodies against a-SMA (1: 2000, #ab5694, Abcam), TGF-b1 (1:2000, sc-130348, Santa Cruz), GAPDH (1:5000, sc-32233, Santa Cruz), coll IaI (1:1000, sc-59772, Santa Cruz), eNOS (1:1000, SC-376751 Santa Cruz), iNOS (1:1000, sc-7271, Santa Cruz), p-SMAD2/3 (1:1000, #PA5-110,155, Invitrogen), and SMAD2/3 (1:1000, #PA5-36,125, Invitrogen). Following incubation, signals were detected using the ChemiDoc Touch imaging system (Bio-Rad) with an enhanced chemiluminescence solution (Bio-Rad, 170–5061). The level of expression was normalized to those of beta-actin using ImageJ software.

### Statistical analysis

The data are expressed as mean ± standard error of mean (SEM). The statistical significance of the differences between two groups or multiple groups was compared by unpaired Student’s t-test or one-way analysis of variance (ANOVA) followed by Tukey’s post hoc multiple comparison test, respectively. P-Values < 0.05, 0.01, 0.001, and 0.0001 were considered statistically significant. All statistical analyses were performed using Prism 9 (GraphPad Software, La Jolla, CA, USA).

## Results

### Bionanomatrix coating characterization

The self-assembled bionanomatrix composed of PA-YK-NO (1 wt.%) was coated on smooth surface silicone implants (10 mm X 10 mm X 1 mm) using an ultrasonic spray coating system (Exactacoat, Sonotek Corporation, NY). With this coating system, we controlled and set up coating pattern, thickness, and time by changing several parameters including flow rate, nozzle movement speed, shaping air, and dwell time [42]. One layer of bionanomatrix coating was applied by one pass of ultrasonic spray. 30 layers of bionanomatrix coating has successfully demonstrated bioactivity in vitro and in vivo on reducing inflammatory responses and enhancing endothelialization in our previous study [42]. Thus, in this study, we characterized 30 layers of bionanomatrix coating on the silicone implants and evaluated its bioactivity on capsular contracture development through in vitro and in vivo analyses. Additionally, higher layers (two times; 60 layers) of bionanomatrix coating on the silicone implants was also evaluated to examine the effects of increased coating layers and NO capacity on capsular contracture development.

**Fig. 1:**
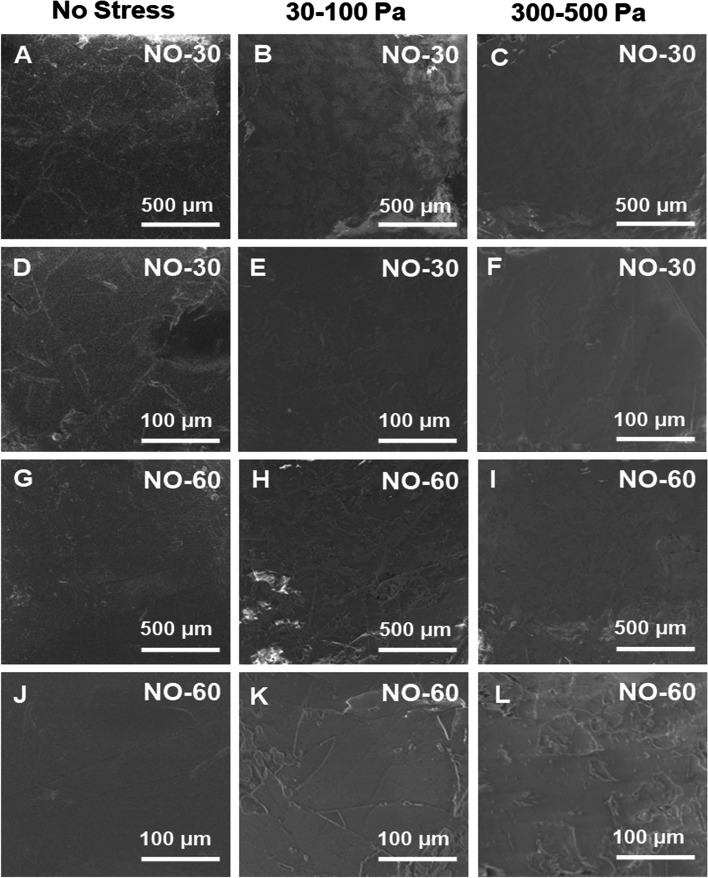
Surface images of 30 and 60 layers of bionanomatrix coated silicone implants mimicking contact shear stress conditions after implantation in the breast by SEM analysis. **A-F** 30 layers of bionanomatrix coating under 0, 30–100 Pa, and 300–500 Pa ranges of contact shear stress. **G-L** 60 layers of bionanomatrix coating under 0, 30–100 Pa, and 300–500 Pa ranges of contact shear stress. NO-30: 30 layers of bionanomatrix coated silicone, NO-60: 60 layers of bionanomatrix coated silicone

Coating surface and uniformity of uncoated, 30, and 60 layers of bionanomatrix coated silicone implants were characterized using scanning electron microscope (SEM). Both 30 and 60 layers of bionanomatrix coatings demonstrated a uniform and smooth surface on the silicone implants (Fig. 1A, D, G, and J; the uncoated silicone surface images are shown in Supplementary Fig. S1).

After breast implantation, silicone implant contact shear stress as a function of geometry and relative size is usually around 100 Pa on the smooth surface silicone implants [56]. At this stress level, epithelial cells can produce pro-inflammatory cytokines and undergo apoptosis [56, 57]. In addition, based on the implant designs, contact shear stress may reach as high as 300–500 Pa range [56]. We tested the coating stability of 30 and 60 layers of bionanomatrix coatings to confirm whether the bionanomatrix coating can be maintained on the silicone implants under these stress conditions. Based on SEM observation, both 30 and 60 layers of bionanomatrix coatings under the stress conditions (30–100 Pa and 300–500 Pa ranges) still demonstrated smoothness and uniformity compared to control groups without stress (Fig. 1). In addition, nanoindentation tests were conducted to assess the changes in properties of bionanomatrix coatings (30 and 60 layers) under stress conditions (300–500 Pa ranges). Before and after shear stress application, the surface modulus and hardness did not show significant changes in all groups (uncoated, 30, and 60 layers of bionanomatrix coatings) (Table 1).

**Table 1:** Nanoindentation analysis of bionanomatrix coated silicone implants

	Cont	NO-30	NO-60
**Modulus at Max Load (GPa)** **(Before Shear Stress of 300–500 Pa)**	0.069 ± 0.002	0.073 ± 0.004	0.071 ± 0.002
**Modulus at Max Load (GPa)** **(After Shear Stress of 300–500 Pa)**	0.070 ± 0.007	0.077 ± 0.002	0.071 ± 0.002
**Hardness at Max Load (GPa)** **(Before Shear Stress of 300–500 Pa)**	0.020 ± 0.002	0.019 ± 0.003	0.021 ± 0.001
**Hardness at Max Load (GPa)** **(After Shear Stress of 300–500 Pa)**	0.020 ± 0.002	0.018 ± 0.001	0.021 ± 0.001

Surface modulus and hardness of uncoated, 30, and 60 layers of bionanomatrix coated silicone implants before and after shear stress application (300–500 Pa) by nanoindentation analysis

**Fig. 2:**
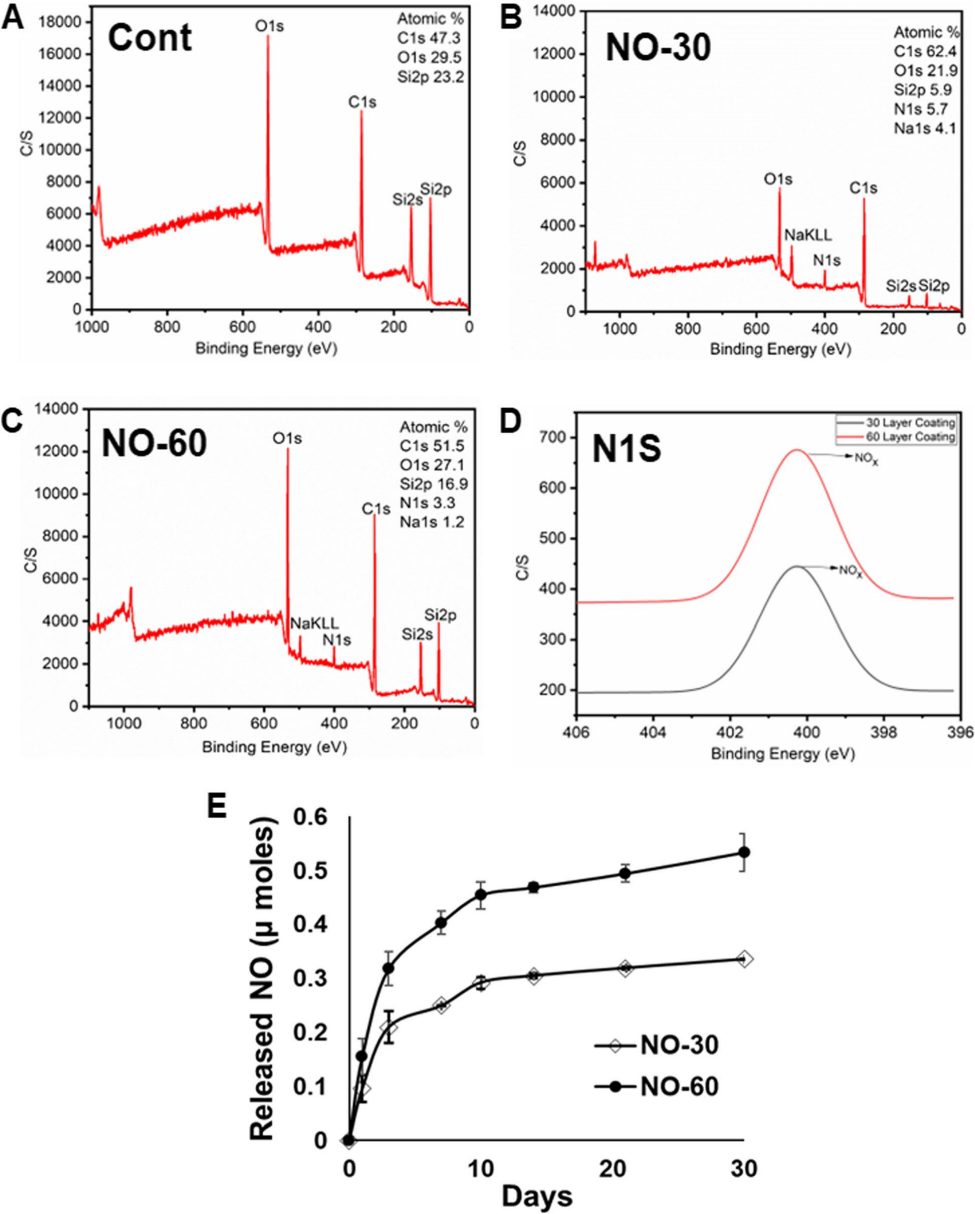
**A-C** Surface analysis of uncoated, 30, and 60 layers of bionanomatrix coated silicone implants using XPS. C/S (count per seconds; intensity) (**D**) High-resolution nitrogen spectra binding energy peak at 404 eV of 30 and 60 layers of bionanomatrix coatings. **E** NO release in to the subcutaneous-mimicking hydrogel for a month analyzed by Total NO assay. Cont: Uncoated silicone, NO-30: 30 layers of bionanomatrix coated silicone, and NO-60: 60 layers of bionanomatrix coated silicone

The X-ray photoelectron spectroscopy (XPS) was utilized to get information on the different surface elements present on the surface of silicone implants. The XPS spectra showed that both 30 and 60 layers of bionanomatrix coated groups presented nitrogen on the surface of the silicone implants, but the nitrogen component was not found in the uncoated control group (Fig. 2A-C). This demonstrated the presence of peptide-based bionanomatrix coating on the silicone implants. In addition, we recorded the high-resolution nitrogen spectra, which showed a binding energy peak at 404 eV for nitrogen (Fig. 2D). This peak was reported to be the characteristic band for NO [68, 69]. This clearly shows the presence of NO on the surface of silicone implants. Additionally, based on the peak intensity, the 60 layers of bionanomatrix coating showed twice as much NO signal compared to the 30 layers.

Next, contact angle measurement was performed on the bionanomatrix coated silicone implants to evaluate whether the bionanomatrix coating increased the wettability of the hydrophobic silicone surface. The 30 and 60 layers of coated silicone implants showed decrease of contact angle values (71.977 ± 1.80 & 53.075 ± 1.50, respectively) compared to the control silicone implants with a contact angle of 93.356 ± 2.30 (Supplementary Fig. S2). The results indicated that the presence of the bionanomatrix increased surface wettability due the hydrophilicity of peptide components in the coating. Additionally, increase of coating layers promoted wettability since the 60 layers of coating showed reduced contact angle compared to the 30 layers of coating.

Nitric oxide (NO) release into a subcutaneous-mimicking hydrogel was evaluated using the Total NO assay [60–62]. The results showed an initial burst release of NO within three days and followed by a sustained release of NO up to one month in both 30 and 60 layers of bionanomatrix coated silicone implants (Fig. 2E). The total release of NO from 60 layers of bionanomatrix coating was about two times greater than the 30 layers of bionanomatrix coating, but the release trend was similar in both groups. Our NO release kinetics and XPS results both demonstrated that increased thickness of bionanomatrix coating is correlated with NO loading capacity. In addition, the NO release kinetics results indicate that the bionanomatrix coated silicone implants are expected to provide NO for at least one month to prevent capsular contracture formation.

### Differentiated monocyte adhesion test on the bionanomatrix coated silicone implants

**Fig. 3:**
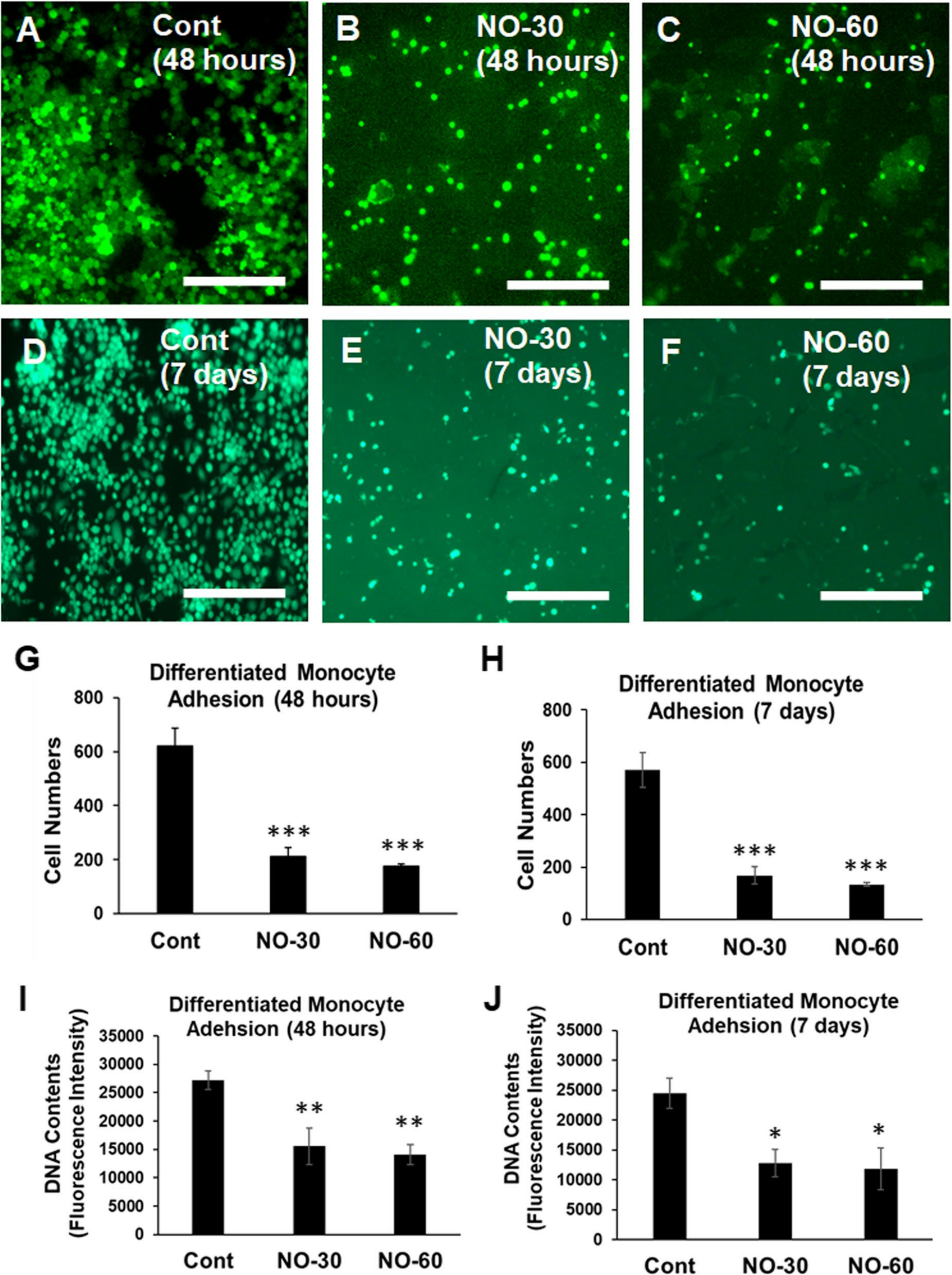
**A-F** Differentiated monocyte adhesion on uncoated, 30, and 60 layers of bionanomatrix coated silicone implants for 48 h and 7 days analyzed by Calcein fluorescent staining. **G-H** Quantification of fluorescent cell number for each group. **I**-**J** Quantification of DNA contents of adhered differentiated monocytes on uncoated, 30, and 60 layers of bionanomatrix coated silicone implants for 48 h and 7 days analyzed by CyQUANT assay. Cont: Uncoated silicone, NO-30: 30 layers of bionanomatrix coated silicone, and NO-60: 60 layers of bionanomatrix coated silicone. White scale bar: 100 μm. *p < 0.05, ***p < 0.01, and ***p < 0.001 vs. uncoated silicone

To evaluate the effects of the bionanomatrix coating on inflammatory cells, adhesion of differentiated monocytes (U937 cells; human monocyte cell-line) on silicone implants was evaluated. PMA (20 ng/ml) differentiated U937 cells with TNF-α (20 ng/ml) were seeded on silicone implants (uncoated, 30, and 60 layers bionanomatrix coated groups) and incubated for 48 h and 7 days. Cell adhesion was analyzed by Calcein staining for live cell visualization and DNA content was quantified using CyQUANT assay. Cell adhesion was significantly reduced on both coated groups compared to the uncoated group at 48 h and 7 days (Fig. 3). There was no significant difference in reduction of cell adhesion between both coated groups. Both time periods (48 h and 7 days) showed similar cell adhesion results. Cell proliferation did not occur, and cell morphology was also maintained after monocyte differentiation, which was consistent with the previous studies [70, 71].

### Biofilm formation assay on the bionanomatrix coated silicone implants

**Fig. 4:**
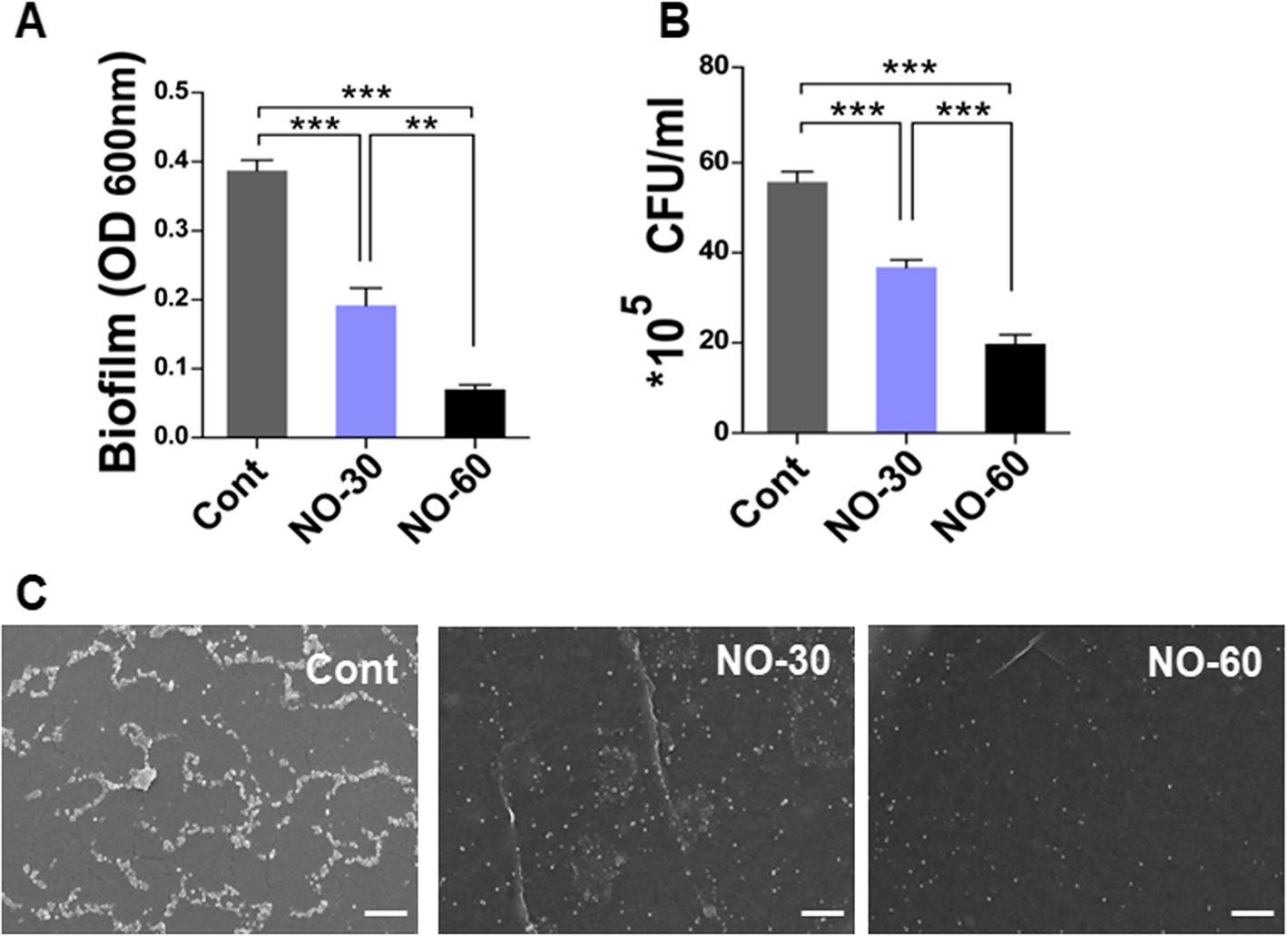
Growth of *S. epidermidis* on bionanomatrix coated silicone implants. *S. epidermidis* were cultured with uncoated, 30, and 60 layers of bionanomatrix coated silicone implants for 48 h. **a** Quantification of biofilm on bionanomatrix coated silicone implants analyzed by measuring the absorbance at 600 nm. **b** CFU analysis of *S. epidermidis* on bionanomatrix coated silicone implants. **c** SEM analysis of biofilm on bionanomatrix coated silicone implants. Cont: Uncoated silicone, NO-30: 30 layers of bionanomatrix coated silicone, and NO-60: 60 layers of bionanomatrix coated silicone. White scale bars: 10 μm. **p < 0.01, ***p < 0.001 between two groups

To evaluate the effects of bionanomatrix coating on biofilm formation, *S. epidermidis*, commonly found on infected silicone implants, was cultured with the bionanomatrix coated (30 and 60 layers of coating) or uncoated silicone implants for 48 h in the 12-well cell culture plate [8, 37, 64]. After incubation, the biofilm was stained with crystal violet and quantified by assessing the absorbance at 600 nm. Colony forming unit (CFU) assay and SEM image analysis were also conducted to assess biofilm formation and viability. The biofilm formation was significantly reduced on both bionanomatrix coated groups compared to the uncoated control group (Fig. 4A). The 60-layered group also showed further reduction of biofilm formation compared to the 30-layered group. The CFU counts and SEM results also showed the same trend with the reduction of biofilm formation on both coated groups compared to the uncoated control group (Fig. 4B-C). The results demonstrated that the NO releasing bionanomatrix coating has antibacterial properties for inhibiting biofilm formation from *S. epidermidis*.

### Effects of bionanomatrix coated silicone on capsule formation

**Fig. 5:**
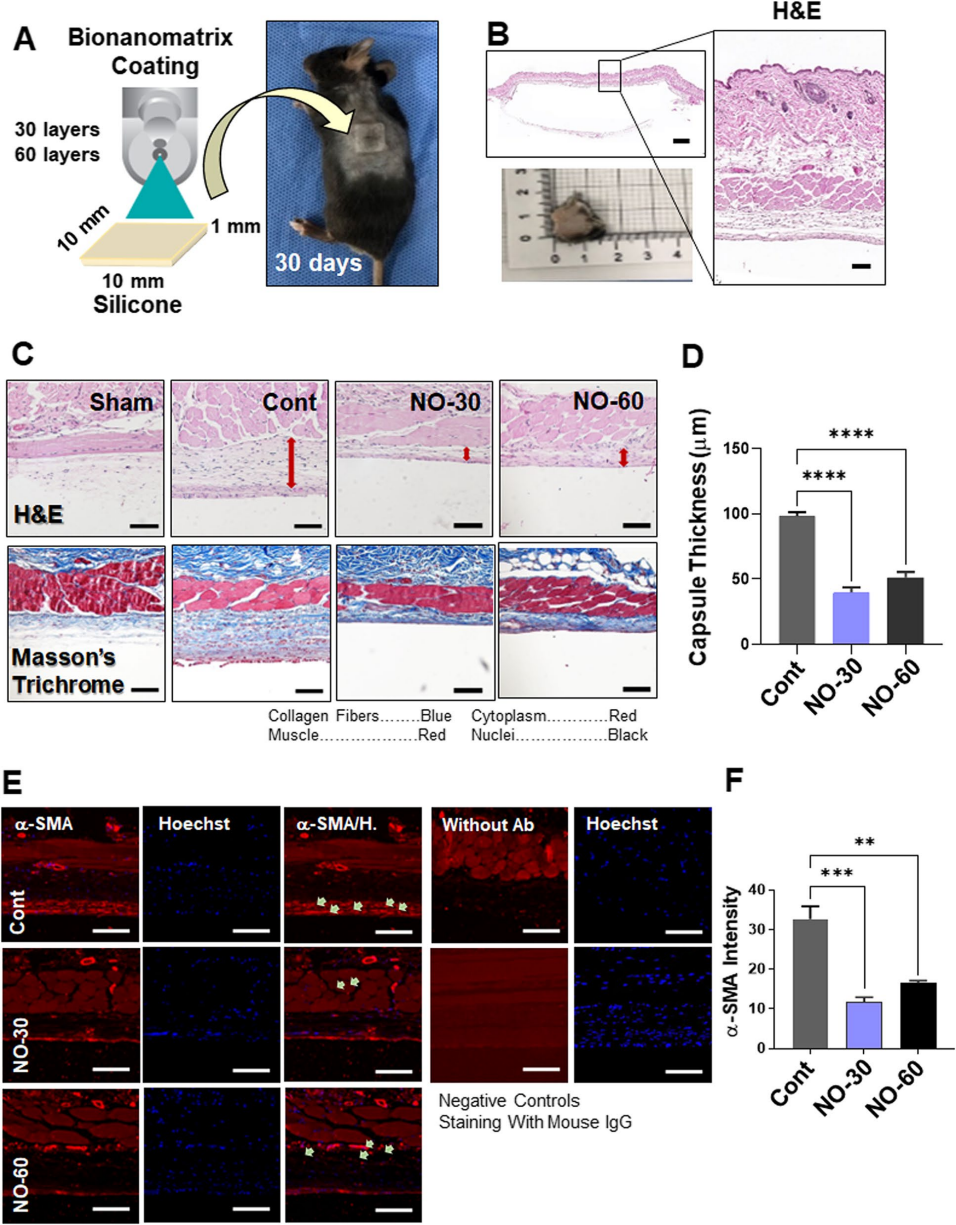
**A** Subcutaneous implantation of bionanomatrix coated silicone implants into the mouse model for a month. **B** Histological analysis of tissues surrounding the implants. Scale bar of left H&E image: 1,000 μm. Scale bar of right H&E image: 100 μm. **C** Analysis of capsule thickness surrounding the implants evaluated by H&E and Masson’s trichrome staining. Scale bar: 100 μm. **D** Quantification of capsule thickness for each group. ****p < 0.0001 between two groups. **E** Evaluation of α-SMA positive cells in the capsules by IHC analysis. Scale bar: 100 μm. **F** Quantification of α-SMA positive cells for each group. **p < 0.01, ***p < 0.001 between two groups. Sham: no implantation (negative control), Cont: Uncoated silicone, NO-30: 30 layers of bionanomatrix coated silicone, and NO-60: 60 layers of bionanomatrix coated silicone

Since NO has been shown to prevent myofibroblast differentiation and fibrotic tissue formation, reduce inflammatory responses, and inhibit bacterial infection or biofilm formation, we hypothesized that the NO releasing bionanomatrix coating would prevent capsular contracture formation by targeting the above multiple factors. To demonstrate this hypothesis, we implanted the bionanomatrix coated silicone implants (30 and 60 layers of coating) subcutaneously in mice for one month (Fig. 5A). After mice sacrifice, histological analyses including hematoxylin and eosin (H&E) staining and Masson’s trichrome staining were conducted to evaluate the development of capsular contracture in the tissues surrounding the implants (Fig. 5B). The capsules in the bionanomatrix coated groups (capsule thickness of 30 and 60 layers: 39.70 ± 8.52 μm and 50.98 ± 8.98 μm, respectively) were significantly thinner compared to the uncoated control group (capsule thickness: 98.08 ± 11.75 μm) (Fig. 5 C and D). Between the coated groups, the 30-layer group showed slightly thinner capsule thickness than the 60-layer group. This result demonstrated that capsule formation was significantly inhibited by the bionanomatrix coating. We also did not observe any foreign body response or serious inflammation in both coated groups. In addition, we evaluated the number of α-smooth muscle actin (SMA) positive cells by immunohistochemistry (IHC) analysis, which represented the myofibroblast content in the capsules (Fig. 5E and F) [37]. α-SMA positive cells in the capsules were significantly lower in the bionanomatrix coated groups compared to the uncoated group. The 30-layer group also had slightly lower α-SMA positive cells compared to the 60-layer group. The result indicated that the bionanomatrix coating suppressed the differentiation of fibroblasts into myofibroblasts, which plays an important role in capsular contracture formation [37]; this data is also consistent with the result of capsule thickness reduction.

**Fig. 6:**
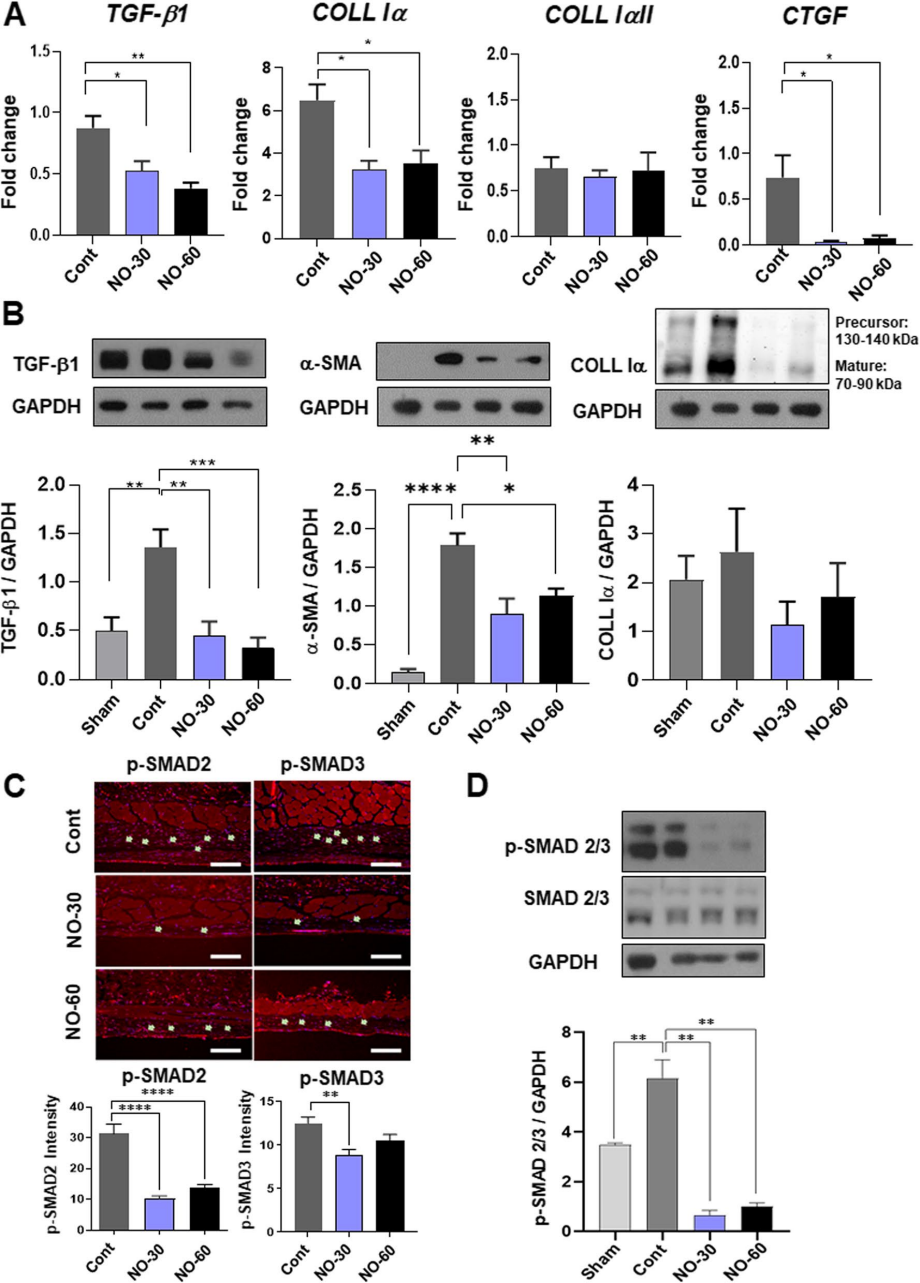
**A** qRT-PCR analysis of TGF-β1, COLL Iα, COLL IαII, and CTGF expressions from the capsule tissues. *p < 0.05 and **p < 0.01 between two groups. **B** Western blot analysis of TGF-β1, α-SMA, and COLL Iα expressions from the capsule tissues. *p < 0.05, **p < 0.01, ***p < 0.001, and ****p < 0.0001 between two groups. **C** IHC analysis of p-SMAD2 and 3 from the capsule tissues. Scale bar: 100 μm. **p < 0.01 and ****p < 0.0001 between two groups. **D** Western blot analysis of p-SMAD2/3 expressions from the capsule tissues. **p < 0.01 between two groups. Sham: no implantation (negative control), Cont: Uncoated silicone, NO-30: 30 layers of bionanomatrix coated silicone, and NO-60: 60 layers of bionanomatrix coated silicone

Transforming growth factor beta (TGF-β) is a key fibrogenic regulatory cytokine for promoting capsular contracture by stimulating fibroblast-to-myofibroblast differentiation and extracellular (ECM) synthesis through TGF-β/p-SMADs signaling cascades [17–19, 72]. To evaluate the effects of bionanomatrix coating on the TGF-β signaling, we analyzed mRNA expression of *TGF-β1*, collagen type Iα *(COLL Iα)* chains*,* and connective tissue growth factor *(CTGF;* mediating stimulatory actions of TGF-β ECM synthesis), in the collected tissues from the capsules using qRT-PCR [73, 74]. *TGF-β1, COLL IαI, and CTGF* were significantly reduced in both bionanomatrix treated groups compared to the uncoated control group (Fig. 6A). *COLL IαII* was not significantly different among all groups. In addition, we confirmed expression of TGF-β1, α-SMA, and COLL Iα at the protein level using western blot analysis. TGF-β1 and α-SMA were significantly decreased in both the bionanomatrix coated groups compared to the uncoated control group (Fig. 6B). COLL Iα was not significantly changed, but both coated groups showed the reduced trend compared to the uncoated group. Furthermore, positive cells for p-SMAD2 and 3 (downstream mediators of the TGF-β/p-SMADs signaling cascades) in the capsules were assessed using IHC. Both coated groups showed significant reduction of p-SMAD2 and 3 positive cells compared to the uncoated group (Fig. 6C). There was no significant difference between both coating groups (30 and 60 layers). The result was also confirmed with western blot analysis of p-SMAD2/3 expression. This expression was significantly reduced in both coated groups compared to the uncoated groups (Fig. 6D), which was also consistent with the results obtained using IHC analysis. Therefore, our in vivo studies demonstrated that the NO releasing bionanomatrix coating significantly inhibited capsular contracture formation by reducing fibroblast to myofibroblast differentiation and fibrous ECM production.

### Effects of bionanomatrix coated silicone on intrinsic NO production

**Fig. 7:**
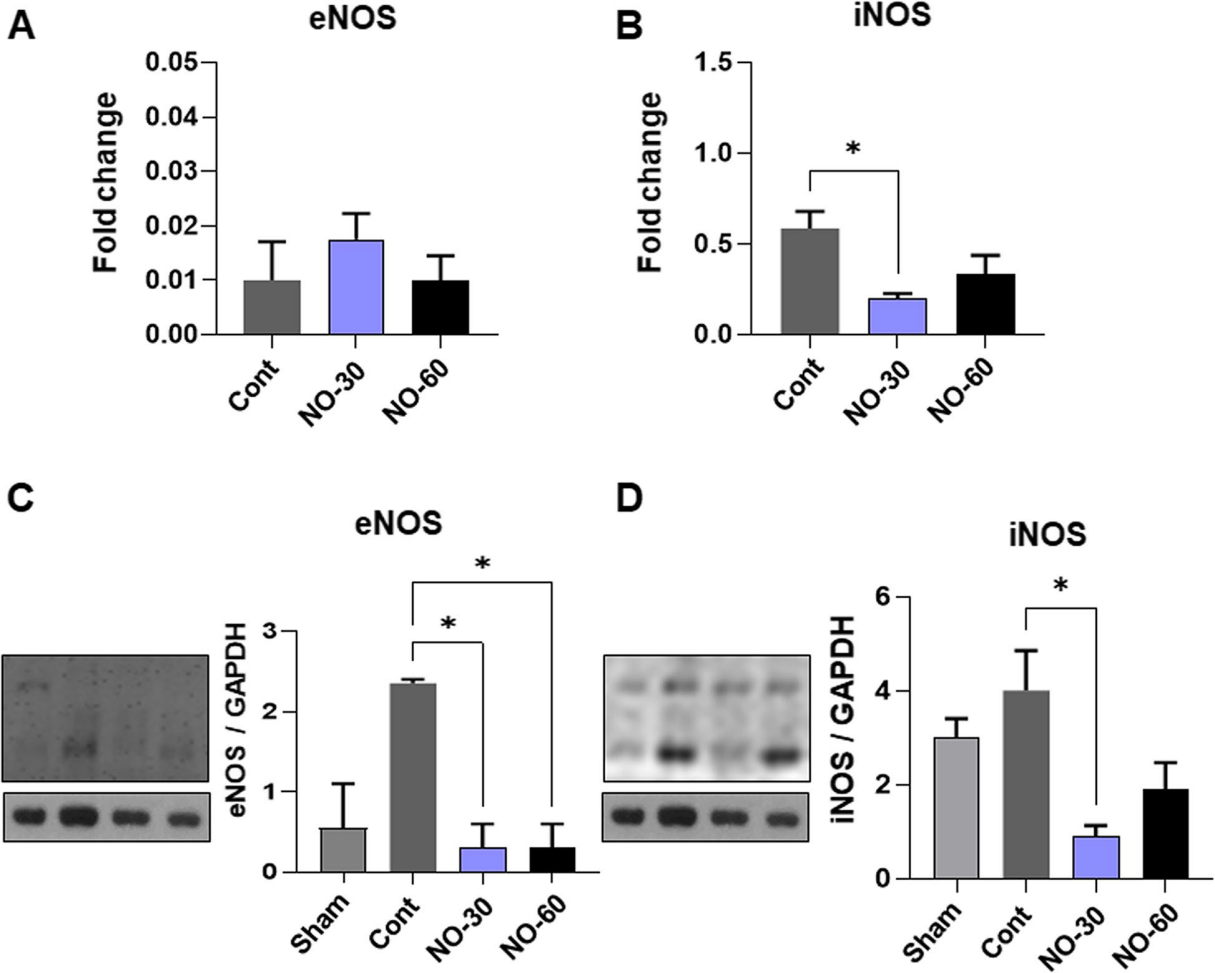
qRT-PCR analysis of (**A**). eNOS and (**B**). iNOS expressions from the capsule tissues. *p < 0.05 between two groups. Western blot analysis of (**C**). eNOS and (**D**). iNOS expressions from the capsule tissues. *p < 0.05 between two groups. Sham: no implantation (negative control), Cont: Uncoated silicone, NO-30: 30 layers of bionanomatrix coated silicone, and NO-60: 60 layers of bionanomatrix coated silicone

To analyze the effects of NO releasing bionanomatrix coating on endogenous NO production from the tissues surrounding the implant, we examined the expression of endothelial and inducible nitric oxide synthases (eNOS and iNOS) using qRT-PCR and western blot analyses. eNOS is typically found in endothelium and related with angiogenesis during healing process [75]. There was no significant difference in mRNA expression of *eNOS* between the three groups, but both bionanomatrix coated groups (30 and 60 layers) showed a significant reduction of eNOS at the protein level compared to the uncoated control group (Fig. 7A and C). iNOS is an important mediator of inflammation in the foreign body response [76, 77]. The 30 layers of bionanomatrix coated group showed a significant reduction of *iNOS* expression at mRNA and protein levels compared to the uncoated control group. The 60-layer coated group also showed the reduced trend of iNOS expression, but it was not significant (Fig. 7 B and D. These results indicated that external NO delivery may reduce endogenous NO production from the tissues surrounding the implants.

### Effects of bionanomatrix coated silicone on inflammation

**Fig. 8:**
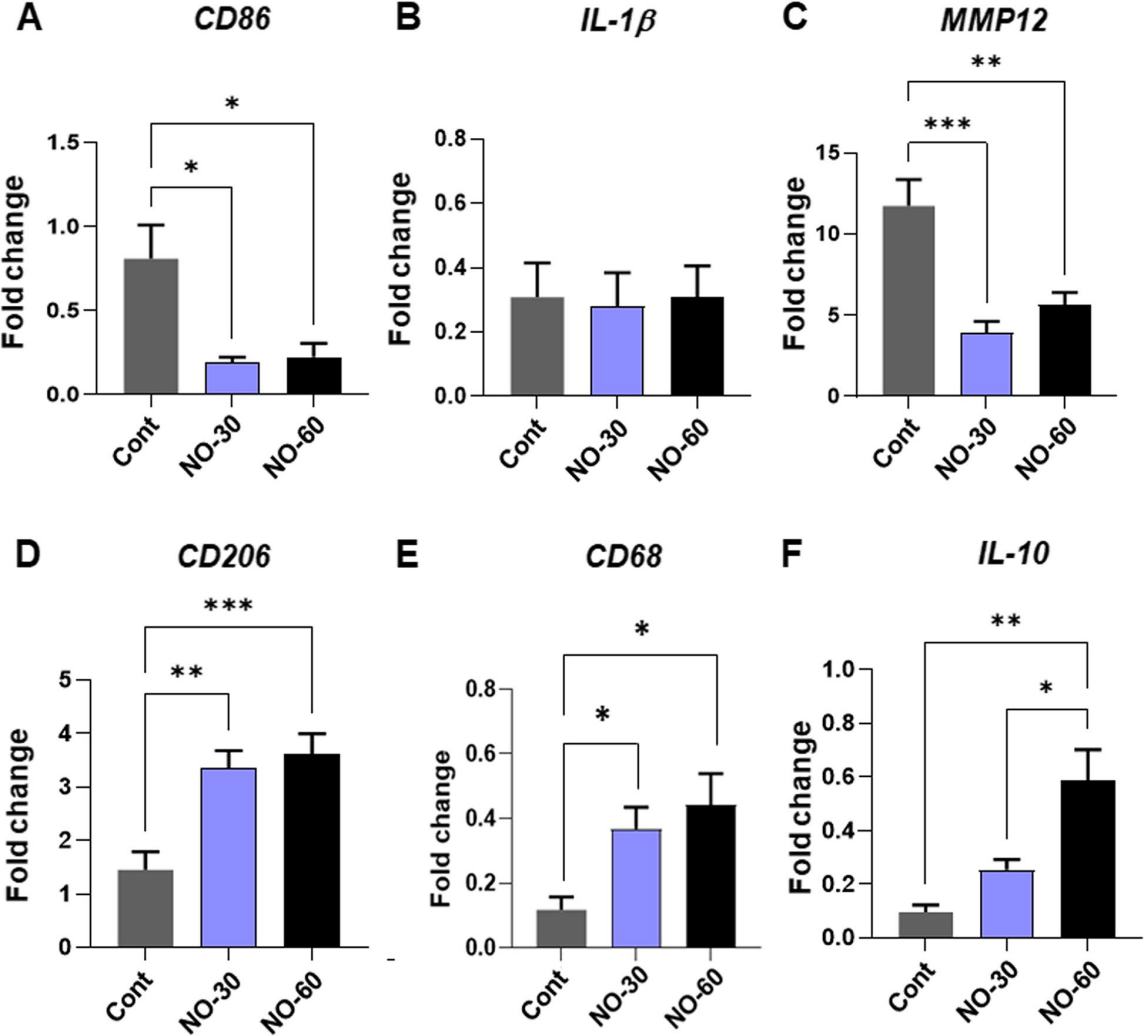
qRT-PCR analysis of (**A**-**C**). CD86, IL-1β, and MMP12 IαII and (**D**-**F**). CD206, CD68, and IL-10 expressions from the capsule tissues. *p < 0.05, **p < 0.01, and ***p < 0.001 between two groups. Cont: Uncoated silicone, NO-30: 30 layers of bionanomatrix coated silicone, and NO-60: 60 layers of bionanomatrix coated silicone

In order to evaluate the effect of the NO releasing bionanomatrix on inflammation, several markers commonly associated with macrophage polarization and activity were assessed 30 days post-implantation using qRT-PCR. *CD86*, a cell surface marker commonly expressed on pro-inflammatory (M1) macrophages, was significantly reduced on both of the bionanomatrix coated groups compared to uncoated silicone implants (Fig. 8A) [78, 79]. Interleukin *(IL)-1β*, a pro-inflammatory cytokine [78, 79], did not show any significant difference between all the groups (Fig. 8B). However, matrix metalloproteinase *(MMP)-12*, a pro-inflammatory marker as well as a regulator of macrophage infiltration and inflammation [80], was significantly reduced for both the bionanomatrix coated groups (Fig. 8C). These results indicate that the NO releasing bionanomatrix reduces M1 macrophage polarization; however, after 30 days implantation, the activity may be low at this point in the healing process. The presence of anti-inflammatory (M2) macrophages was also assessed. Notably, *CD206* and *CD68*, surface markers commonly expressed on M2 macrophages [78, 79, 81], were significantly increased for both the bionanomatrix coated groups (Fig. 8 D and E). Additionally, anti-inflammatory cytokine, *IL10* [78, 79], was significantly increased for the bionanomatrix coated groups (Fig. 8F).

## Discussion

Capsular contracture is a serious complication of silicone implantation mostly caused by an excessive foreign body reaction. This results in fibrotic tissue formation on the implant which may lead to implant deformation. Currently, a target mechanism for capsule formation has not been clearly identified. Myofibroblast differentiation and ECM production through the TGF-β/SMADs signaling cascades are considered a crucial factor for the development of capsular contracture. Biofilm formation and chronic inflammation on silicone implants may also promote fibrotic capsule formation. Although various treatments including surgical intervention and medication have been used for capsular contracture therapy, there are no approaches to target the multi-faceted mechanisms for inhibiting capsular contracture formation.

Our multi-targeting NO releasing bionanomatrix coating may provide a new therapeutic approach to reduce the development of capsular contracture by targeting myofibroblast differentiation, inflammatory responses, and infections. NO has been known to reduce myofibroblast differentiation, inflammatory responses, biofilm formation, and fibrotic tissue formation. The self-assembled PA based bionanomatrix coating enables a sustained delivery of NO by utilizing bioactive peptide sequences including NO producing donor and enzyme-mediated degradation site. In this study, the bionanomatrix coating was uniformly and smoothly applied to the silicone implants, and the coating demonstrated mechanical stability under shear stress conditions, which was in the range of increased contact shear stress based on typical silicone implant environmental conditions [56, 57]. These results indicated that our bionanomatrix coating can still be stable without cracking or detachment after implantation. In addition, the bionanomatrix coating showed a sustained release of NO to the subcutaneous-mimicking hydrogel model for at least over a month. This early period is critical since capsular contracture formation is mostly caused by an excessive foreign body reaction that is predominant 4 weeks after implantation until the implants are settled during the healing process [82, 83]. Thus, we expect the bionanomatrix coating will effectively inhibit capsular contracture formation by delivery of NO.

Also, we tested adhesion of differentiated monocytes on the bionanomatrix coated silicone implants in vitro*,* and cell adhesion was significantly reduced on the bionanomatrix coated group compared to the uncoated group. This indicates that the bionanomatrix coating may reduce inflammatory responses on the silicone implants since macrophages play an important role in the foreign body responses leading to capsular contracture [13, 15]. Furthermore, the bionanomatrix coated silicone implants showed a significant reduction of biofilm formation of *S. epidermidis*, a commonly found bacteria on infected silicone implants, in vitro. The antibacterial properties of bionanomatrix have also been demonstrated in our previous results where the NO releasing bionanomatrix inhibited bacterial growth and biofilm formation of endodontic microorganisms including *E. faecalis*, *T. denticola*, and clinical samples [43].

We then evaluated the hypothesis whether the NO releasing bionanomatrix coating reduced capsular contracture formation on silicone implants using a subcutaneous mouse model. The bionanomatrix coated silicone implant groups showed a significant reduction of capsule thickness and expression of myofibroblast differentiation marker, α-SMA compared to the uncoated group after one month subcutaneous implantation. Additionally, no foreign body response or any serious inflammation was observed. The bionanomatrix coated group also showed a reduction in the expressions of mediators of TGF-β/p-SMADs signaling cascade which is important for promoting fibroblast to myofibroblast differentiation and ECM production. Therefore, the bionanomatrix coating demonstrated a significant reduction in capsular contracture formation on silicone implants by decreasing fibroblast to myofibroblast differentiation and fibrous ECM production. This can be associated with the NO effects on the TGF-β/SMADs signaling cascades. Our results are also consistent with the previous studies that NO reduced fibroblast to myofibroblast differentiation and collagen synthesis through TGF-β signaling [49, 50, 84].

We also analyzed the expression of eNOS and iNOS to examine the effects of NO releasing bionanomatrix coating on endogenous NO production from the tissues surrounding the implant. Our results showed an external NO delivery may reduce endogenous NO production. This may be attributed to the fact that delivery of NO might promote the healing process and this early adaptation could reduce the foreign body reaction and intrinsic NO production. These findings require further investigation. In addition, the bionanomatrix coating reduced the expressions of some pro-inflammatory macrophage polarization (M1) markers but increased anti-inflammatory markers (M2) in the implant surrounding tissues. These results suggest that the NO releasing bionanomatrix may promote M2 macrophage polarization and activity which may enhance healing, reduce inflammation, and thereby inhibit capsular contracture development.

Overall, our in vitro and in vivo studies demonstrated the feasibility that the NO releasing bionanomatrix coating significantly inhibits capsular contracture development on silicone implants by multi-targeted strategy. The application of this bionanomatrix coating can also be achieved to various implantable biomedical devices to reduce fibrotic tissue formation as well as to promote biocompatibility and healing process after implantation. Since the presence of undetected subclinical infection may increase the risks of capsular contracture, we may further investigate the treatment of bionanomatrix coated silicone implants in infected animal models to examine the effects bionanomatrix coating on infection and the development of capsular contracture over a longer-time period as a future study.

## Conclusions

In summary, we characterized the multi-targeting NO releasing bionanomatrix coating on the silicone implants and successfully demonstrated the reduction of capsular contracture formation in vitro and in vivo. We tested the 30 and 60 layers of bionanomatrix coatings to evaluate the effects of increased NO capacity by increased coating layers. First, both layers of bionanomatrix were uniformly and smoothly coated on the silicone implants and showed mechanical stability up to 500 Pa of contact shear stress which mimics the environment of high levels of shear stress after implantation in the breast. In addition, both bionanomatrix coatings presented a sustained release of NO to the subcutaneous-mimicking hydrogel model for over one month. Bionanomatrix coatings also demonstrated the reduction of differentiated monocytes and *S. epidermidis* biofilm formation on the surface of coated silicones in vitro. Both bionanomatrix coated silicone implant groups were applied to the subcutaneous mouse model for a month in vivo. Both coated groups significantly reduced capsule thickness surrounding the implants by reducing fibroblast to myofibroblast differentiation and fibrous ECM production through the TGF-β/SMADs signaling cascades. There was no significant difference in reduction of capsule thickness and myofibroblast differentiation between both coating groups, indicating that the 30 layers of bionanomatrix coating was sufficient in reducing capsular contracture formation. Also, both coated groups demonstrated biocompatibility and advanced the healing process by promoting M2 macrophage polarization. Therefore, based on the results from this study, the NO releasing bionanomatrix coating may provide great potential to reduce capsular contracture formation on the silicone implants as well as other various implantable devices without significantly modifying the implant itself.

## Supplementary Information

**Additional file 1: Fig. S1.** Scanning electron microscopyimage of uncoated silicone surface. **Fig. S2.** Contact angle measurement ofuncoated,30-layer bionanomatrix coated, and60-layer bionanomatrix coatedsilicone implants.As per journal requirements, every additional file must have a corresponding caption. In this regard, please be informed that the caption was taken from the additional e-file itself. Please advise if the action taken is appropriate and amend if necessary.There is no problem with captions.

## Data Availability

All data and materials are available upon request.

## Abbreviations

TGF-β: Transforming growth factor beta
NO: Nitric oxide
ECM: Extracellular matrix
p-SMADs: Phosphorylated-small mothers against decapentaplegics
TNF-α: Tumor necrosis factor alpha
PA: Peptide amphiphile
MMP: Matrix metalloproteinase
SEM: Scanning electron microscope
XPS: X-ray photoelectron spectroscopy
PMA: Phorbol 12-myristate 13-acetate
TSB: Tryptic soy broth
α-SMA: Alpha smooth muscle actin
eNOS: Endothelial nitric oxide synthase
iNOS: Inducible nitric oxide synthase
qRT-PCR: Quantitative real-time polymerase chain reaction
PVDF: Polyvinylidene fluoride
H&E: Hematoxylin and eosin
IHC: Immunohistochemistry
COLL Iα: Collagen type Iα
CTGF: Connective tissue growth factor
IL: Interleukin

## References

[1] Headon H, Kasem A, Mokbel K (2015). Capsular contracture after breast augmentation: an update for clinical practice. Arch Plast Surg.

[2] Siggelkow W, Faridi A, Spiritus K, Klinge U, Rath W, Klosterhalfen B (2003). Histological analysis of silicone breast implant capsules and correlation with capsular contracture. Biomaterials.

[3] Papaconstantinou A, Koletsa T, Demiri E, Gasteratos K, Tzorakoleftheraki SE, Pavlidis L (2020). Nonsurgical treatment of capsular contracture: Review of clinical studies. J Int Med Res.

[4] Lista F, Ahmad J (2013). Evidence-based medicine: augmentation mammaplasty. Plast Reconstr Surg.

[5] Coombs DM, Grover R, Prassinos A, Gurunluoglu R (2019). Breast augmentation surgery: Clinical considerations. Cleve Clin J Med.

[6] Virden CP, Dobke MK, Stein P, Parsons CL, Frank DH (1992). Subclinical infection of the silicone breast implant surface as a possible cause of capsular contracture. Aesthetic Plast Surg.

[7] Tamboto H, Vickery K, Deva AK. Subclinical (Biofilm) infection causes capsular contracture in a porcine model following augmentation mammaplasty. Plastic Reconstruct Surg. 2010;126(3):835–42. 10.1097/PRS.0b013e3181e3b456 20811216

[8] Danino MA, Nizard N, Paek LS, Govshievich A, Giot JP (2017). Do Bacteria and Biofilm Play a Role in Double-Capsule Formation around Macrotextured Implants?. Plast Reconstr Surg.

[9] Hu H, Jacombs A, Vickery K, Merten SL, Pennington DG, Deva AK (2015). Chronic biofilm infection in breast implants is associated with an increased T-cell lymphocytic infiltrate: implications for breast implant-associated lymphoma. Plast Reconstr Surg.

[10] Rieger UM, Mesina J, Kalbermatten DF, Haug M, Frey HP, Pico R (2013). Bacterial biofilms and capsular contracture in patients with breast implants. Br J Surg.

[11] Galdiero M, Larocca F, Iovene MR, Francesca M, Pieretti G, D'Oriano V (2018). Microbial Evaluation in Capsular Contracture of Breast Implants. Plast Reconstr Surg.

[12] Wolfram D, Rainer C, Niederegger H, Piza H, Wick G (2004). Cellular and molecular composition of fibrous capsules formed around silicone breast implants with special focus on local immune reactions. J Autoimmun.

[13] Tan KT, Wijeratne D, Shih B, Baildam AD, Bayat A (2010). Tumour necrosis factor-α expression is associated with increased severity of periprosthetic breast capsular contracture. Eur Surg Res.

[14] Prantl L, Angele P, Schreml S, Ulrich D, Poppl N, Eisenmann-Klein M (2006). Determination of serum fibrosis indexes in patients with capsular contracture after augmentation with smooth silicone gel implants. Plast Reconstr Surg.

[15] Brazin J, Malliaris S, Groh B, Mehrara B, Hidalgo D, Otterburn D (2014). Mast cells in the periprosthetic breast capsule. Aesthetic Plast Surg.

[16] Moyer KE, Ehrlich HP (2013). Capsular contracture after breast reconstruction: collagen fiber orientation and organization. Plast Reconstr Surg.

[17] Katzel EB, Koltz PF, Tierney R, Williams JP, Awad HA, O'Keefe RJ (2011). The impact of Smad3 loss of function on TGF-beta signaling and radiation-induced capsular contracture. Plast Reconstr Surg.

[18] Sime PJ, Xing Z, Graham FL, Csaky KG, Gauldie J (1997). Adenovector-mediated gene transfer of active transforming growth factor-beta1 induces prolonged severe fibrosis in rat lung. J Clin Invest.

[19] Border WA, Noble NA (1994). Transforming growth factor beta in tissue fibrosis. N Engl J Med.

[20] Hwang K, Sim HB, Huan F, Kim DJ (2010). Myofibroblasts and capsular tissue tension in breast capsular contracture. Aesthetic Plast Surg.

[21] Baker JL, Chandler ML, LeVier RR (1981). Occurrence and activity of myofibroblasts in human capsular tissue surrounding mammary implants. Plast Reconstr Surg.

[22] Wan D, Rohrich RJ (2016). Revisiting the Management of Capsular Contracture in Breast Augmentation: A Systematic Review. Plast Reconstr Surg.

[23] Brown T. Plane Change Vs Capsulotomy: A comparison of treatments for capsular contraction in breast augmentation using the subfascial plane. Aesthetic Plast Surg. 2020;45(3):845–50. 10.1007/s00266-020-02010-8 33078211

[24] Graf R, Ascenço ASK, Freitas RdS, Balbinot P, Peressutti C, Costa DFB (2015). Prevention of capsular contracture using leukotriene antagonists. Plast Reconstr Surg..

[25] Huang CK, Handel N (2010). Effects of singulair (Montelukast) treatment for capsular contracture. Aesthetic Surg J.

[26] Scuderi N, Mazzocchi M, Fioramonti P, Bistoni G (2006). The effects of zafirlukast on capsular contracture: preliminary report. Aesthetic Plast Surg.

[27] Adams WP, Conner WC, Barton FE, Rohrich RJ (2000). Optimizing breast pocket irrigation: an in vitro study and clinical implications. Plast Reconstr Surg..

[28] Bai L, Peng X, Liu Y, Sun Y, Wang X, Wang X (2018). Clinical analysis of 86 botulism cases caused by cosmetic injection of botulinum toxin (BoNT). Medicine (Baltimore).

[29] Anselme K, Bigerelle M (2011). Role of materials surface topography on mammalian cell response. Int Mater Rev.

[30] Jeon HJ, Kang M, Lee JS, Kang J, Kim EA, Jin HK (2022). Impact on capsule formation for three different types of implant surface tomography. Sci Rep.

[31] Quesada AE, Medeiros LJ, Clemens MW, Ferrufino-Schmidt MC, Pina-Oviedo S, Miranda RN (2019). Breast implant-associated anaplastic large cell lymphoma: a review. Mod Pathol.

[32] Sharma B, Jurgensen-Rauch A, Pace E, Attygalle AD, Sharma R, Bommier C (2020). Breast implant–associated anaplastic large cell lymphoma: review and multiparametric imaging paradigms. Radiographics.

[33] Castel N, Soon-Sutton T, Deptula P, Flaherty A, Parsa FD (2015). Polyurethane-coated breast implants revisited: a 30-year follow-up. Arch Plast Surg.

[34] Handel N, Gutierrez J (2006). Long-term safety and efficacy of polyurethane foam-covered breast implants. Aesthet Surg J.

[35] Yalanis GC, Liu EW, Cheng HT (2015). Efficacy and safety of povidone-iodine irrigation in reducing the risk of capsular contracture in aesthetic breast augmentation: a systematic review and meta-analysis. Plast Reconstr Surg.

[36] Barnea Y, Hammond DC, Geffen Y, Navon-Venezia S, Goldberg K (2018). Plasma activation of a breast implant shell in conjunction with antibacterial irrigants enhances antibacterial activity. Aesthetic Surg J.

[37] Shin CM, Cho S, Kim DH, Ha Y, Shin HJ, Shin N, et al. Zwitterionic polydopamine coatings suppress silicone implant-induced capsule formation. Biomater Sci. 2021;9:3425–32. 10.1039/d0bm02215b 33949402

[38] Park SO, Han J, Minn KW, Jin US (2013). Prevention of capsular contracture with Guardix-SG® after silicone implant insertion. Aesthetic Plast Surg.

[39] Lee SG, Lee SD, Kim MK, Ryu WS, Jung SP, Kim S (2015). Effect of Antiadhesion barrier solution and fibrin on capsular formation after silicone implant insertion in a white rat model. Aesthetic Plast Surg.

[40] Kushwaha M, Anderson J, Minor W, Andukuri A, Bosworth C, Lancaster J (2010). Native endothelium mimicking self-assembled nanomatrix for cardiovascular devices. Biomaterials.

[41] Alexander GC, Hwang PTJ, Chen J, Kim J, Brott BC, Yoon YS (2018). Nanomatrix coated stent enhances endothelialization but reduces platelet, smooth muscle cell, and monocyte adhesion under physiologic conditions. ACS Biomater Sci Eng.

[42] Hwang PTJ, Sherwood JA, Millican RC, Bobba PS, Lynd TO, Garner JN (2021). Endothelium-mimicking nanomatrix coating to enhance endothelialization after left atrial appendage closure device implantation. ACS Appl Bio Mater.

[43] Moon CY, Nam OH, Kim M, Lee HS, Kaushik SN, Cruz Walma DA (2018). Effects of the nitric oxide releasing biomimetic nanomatrix gel on pulp-dentin regeneration: Pilot study. PLoS ONE.

[44] Jun H-W, Yuwono V, Paramonov SE, Hartgerink JD (2005). Enzyme-Mediated Degradation of Peptide-Amphiphile Nanofiber Networks. Adv Mater.

[45] Anderson JM, Andukuri A, Lim DJ, Jun H-W (2009). Modulating the gelation properties of self-assembling peptide amphiphiles. ACS Nano.

[46] Andukuri A, Kushwaha M, Tambralli A, Anderson JM, Dean DR, Berry JL (2011). A hybrid biomimetic nanomatrix composed of electrospun polycaprolactone and bioactive peptide amphiphiles for cardiovascular implants. Acta Biomater.

[47] Bath P, Coleman C, Gordon A, Lim W, Webb A. Nitric oxide for the prevention and treatment of viral, bacterial, protozoal and fungal infections. F1000Res. 2021;10:536–69. 10.12688/f1000research.51270.1 PMC9171293 35685687

[48] Schairer DO, Chouake JS, Nosanchuk JD, Friedman AJ (2012). The potential of nitric oxide releasing therapies as antimicrobial agents. Virulence.

[49] Vernet D, Ferrini MG, Valente EG, Magee TR, Bou-Gharios G, Rajfer J (2002). Effect of nitric oxide on the differentiation of fibroblasts into myofibroblasts in the Peyronie's fibrotic plaque and in its rat model. Nitric Oxide.

[50] Mookerjee I, Hewitson TD, Halls ML, Summers RJ, Mathai ML, Bathgate RA (2009). Relaxin inhibits renal myofibroblast differentiation via RXFP1, the nitric oxide pathway, and Smad2. FASEB J.

[51] Alexander GC, Vines JB, Hwang P, Kim T, Kim JA, Brott BC (2016). Novel Multifunctional Nanomatrix Reduces Inflammation in Dynamic Conditions in Vitro and Dilates Arteries ex Vivo. ACS Appl Mater Interfaces.

[52] Andukuri A, Min I, Hwang P, Alexander G, Marshall LE, Berry JL (2014). Evaluation of the effect of expansion and shear stress on a self-assembled endothelium mimicking nanomatrix coating for drug eluting stents in vitro and in vivo. Biofabrication.

[53] Jun HW, Paramonov SE, Dong H, Forraz N, McGuckin C, Hartgerink JD (2008). Tuning the mechanical and bioresponsive properties of peptide-amphiphile nanofiber networks. J Biomater Sci Polym Ed.

[54] Hwang PTJ, Shah DK, Garcia JA, Alexander GC, Lim DJ, Cui W (2017). Encapsulation of human islets using a biomimetic self-assembled nanomatrix gel for protection against cellular inflammatory responses. ACS Biomater Sci Eng.

[55] Somarathna M, Hwang PT, Millican RC, Alexander GC, Isayeva-Waldrop T, Sherwood JA (2022). Nitric oxide releasing nanomatrix gel treatment inhibits venous intimal hyperplasia and improves vascular remodeling in a rodent arteriovenous fistula. Biomaterials.

[56] Pitenis AA, Sawyer WG (2020). Soft textured implants: roughness, friction, and the complications. Biotribology.

[57] Pitenis AA, Urueña JM, Hart SM, O’Bryan CS, Marshall SL, Levings PP (2018). Friction-Induced Inflammation. Tribol Lett.

[58] Hwang PTJ, Lim D-J, Fee T, Alexander GC, Tambralli A, Andukuri A (2016). A bio-inspired hybrid nanosack for graft vascularization at the omentum. Acta Biomater.

[59] Vijayan VM, Tucker BS, Hwang PTJ, Bobba PS, Jun HW, Catledge SA (2020). Non-equilibrium organosilane plasma polymerization for modulating the surface of PTFE towards potential blood contact applications. J Mater Chem B.

[60] Leung DH, Kapoor Y, Alleyne C, Walsh E, Leithead A, Habulihaz B (2017). Development of a convenient in vitro gel diffusion model for predicting the In Vivo performance of subcutaneous parenteral formulations of large and small molecules. AAPS PharmSciTech.

[61] Ye F, Larsen SW, Yaghmur A, Jensen H, Larsen C, Ostergaard J (2012). Drug release into hydrogel-based subcutaneous surrogates studied by UV imaging. J Pharm Biomed Anal.

[62] Li Z, Mu H, Weng Larsen S, Jensen H, Østergaard J (2021). An in vitro gel-based system for characterizing and predicting the long-term performance of PLGA in situ forming implants. Int J Pharm.

[63] Hoffmann D, Pilotte L, Stroobant V, Van den Eynde BJ (2019). Induction of tryptophan 2,3-dioxygenase expression in human monocytic leukemia/lymphoma cell lines THP-1 and U937. Int J Tryptophan Res..

[64] Kwasny SM, Opperman TJ (2010). Static biofilm cultures of Gram-positive pathogens grown in a microtiter format used for anti-biofilm drug discovery. Curr Protoc Pharmacol..

[65] Chau Nguyen TT, Shin CM, Lee SJ, Koh ES, Kwon HH, Park H (2022). Ultrathin nanostructured films of hyaluronic acid and functionalized β-cyclodextrin polymer suppress bacterial infection and capsular formation of medical silicone implants. Biomacromol.

[66] Lee SD, Yi MH, Kim DW, Lee Y, Choi Y, Oh SH (2016). The effect of botulinum neurotoxin type A on capsule formation around silicone implants: the in vivo and in vitro study. Int Wound J.

[67] Schmittgen TD, Livak KJ (2008). Analyzing real-time PCR data by the comparative C(T) method. Nat Protoc.

[68] Dubey M, Bernasek SL, Schwartz J (2007). Highly sensitive nitric oxide detection using X-ray photoelectron spectroscopy. J Am Chem Soc.

[69] Abujarada S, Walton AS, Thomas AG, Chohan UK, Koehler SPK (2019). Adsorption site, orientation and alignment of NO adsorbed on Au(100) using 3D-velocity map imaging, X-ray photoelectron spectroscopy and density functional theory. Phys Chem Chem Phys.

[70] Hattori T, Pack M, Bougnoux P, Chang ZL, Hoffman T (1983). Interferon-induced differentiation of U937 cells. Comparison with other agents that promote differentiation of human myeloid or monocytelike cell lines. J Clin Invest..

[71] Lee YH, Lee HJ, Lee SJ, Min DS, Baek SH, Kim YS (1995). Down-regulation of phospholipase C-gamma 1 during the differentiation of U937 cells. FEBS Lett.

[72] Kim S, Ahn M, Piao Y, Ha Y, Choi DK, Yi MH (2016). Effect of Botulinum toxin type A on TGF-β/Smad pathway signaling: implications for silicone-induced capsule formation. Plast Reconstr Surg.

[73] Walraven M, Akershoek JJ, Beelen RH, Ulrich MM (2017). In vitro cultured fetal fibroblasts have myofibroblast-associated characteristics and produce a fibrotic-like environment upon stimulation with TGF-β1: Is there a thin line between fetal scarless healing and fibrosis?. Arch Dermatol Res.

[74] Ihn H (2002). Pathogenesis of fibrosis: role of TGF-beta and CTGF. Curr Opin Rheumatol.

[75] Duda DG, Fukumura D, Jain RK (2004). Role of eNOS in neovascularization: NO for endothelial progenitor cells. Trends Mol Med.

[76] Zamora R, Vodovotz Y, Billiar TR (2000). Inducible nitric oxide synthase and inflammatory diseases. Mol Med.

[77] Xue Q, Yan Y, Zhang R, Xiong H (2018). Regulation of iNOS on immune cells and its role in diseases. Int J Mol Sci.

[78] Poltavets AS, Vishnyakova PA, Elchaninov AV, Sukhikh GT, Fatkhudinov TK (2020). Macrophage modification strategies for efficient cell therapy. Cells.

[79] Orecchioni M, Ghosheh Y, Pramod AB, Ley K (2019). Macrophage polarization: different gene signatures in M1(LPS+) vs. Classically and M2(LPS-) vs. Alternatively activated macrophages. Front Immunol..

[80] Aristorena M, Gallardo-Vara E, Vicen M, de Las C-E, Ojeda-Fernandez L, Nieto C (2019). MMP-12, secreted by pro-inflammatory macrophages, targets endoglin in human macrophages and endothelial cells. Int J Mol Sci.

[81] Klar AS, Michalak-Mićka K, Biedermann T, Simmen-Meuli C, Reichmann E, Meuli M (2018). Characterization of M1 and M2 polarization of macrophages in vascularized human dermo-epidermal skin substitutes in vivo. Pediatr Surg Int.

[82] Shin BH, Kim BH, Kim S, Lee K, Choy YB, Heo CY (2018). Silicone breast implant modification review: overcoming capsular contracture. Biomaterials Research.

[83] Park JU, Ham J, Kim S, Seo JH, Kim SH, Lee S (2014). Alleviation of capsular formations on silicone implants in rats using biomembrane-mimicking coatings. Acta Biomater.

[84] Kanno Y, Into T, Lowenstein CJ, Matsushita K (2008). Nitric oxide regulates vascular calcification by interfering with TGF- signalling. Cardiovasc Res.

