# Supplementary material for: A multi-targeting bionanomatrix coating to reduce capsular contracture development on silicone implants
Source: Biomater Res. 2023 Apr 22;27:34. doi: 10.1186/s40824-023-00378-7 (PMC10122329; doi:10.1186/s40824-023-00378-7)
Supplement: Supplementary file 1 — Additional file 1: Fig. S1. Scanning electron microscopyimage of uncoated silicone surface. Fig. S2. Contact angle measurement ofuncoated,30-layer bionanomatrix coated, and60-layer bionanomatrix coatedsilicone implants.As per journal requirements, every additional file must have a corresponding caption. In this regard, please be informed that the caption was taken from the additional e-file itself. Please advise if the action taken is appropriate and amend if necessary.There is no problem with captions. [file 40824_2023_378_MOESM1_ESM.pdf]

Supplementary Information

A Multi-targeting Bionanomatrix Coating to Reduce Capsular Contracture Development on Silicone Implants

Patrick T.J. Hwang[1,2#], Chung Min Shin[3#], Jennifer A. Sherwood[1], Dong Ho Kim[4], Vineeth M. Vijayan[5], Krishna C. Josyula[2], Reid C. Millican[1], Donald Ho[6], Brigitta C. Brott[1,7], Vinoy Thomas[8], Chul Hee Choi[4], Sang-Ha Oh[3], Dong Woon Kim[9], and Ho-Wook Jun[1,2*]*

[1]Endomimetics, LLC, Birmingham, Alabama, 35242, USA

[2]Department of Biomedical Engineering, University of Alabama at Birmingham, Alabama, 35294, USA

[3]Department of Plastic and Reconstructive Surgery, Chungnam National University College of Medicine, Daejeon, 35015, Republic of Korea

[4]Department of Microbiology, Chungnam National University College of Medicine, Daejeon, 35015, Republic of Korea

[5]Department of Biomedical Engineering, Alabama State University, Alabama, 36104, USA

[6]Department of Pediatric Dentistry, University of Alabama at Birmingham, Alabama, 35294, USA

[7]Department of Medicine and Division of Cardiovascular Disease, University of Alabama at Birmingham, Alabama, 35233, USA

[8]Department of Material Science and Engineering, University of Alabama at Birmingham, Alabama, 35294, USA

[9.]Department of Anatomy and Cell Biology, Brain Research Institute, Chungnam National University College of Medicine, Daejeon, 35015, Republic of Korea

[#]These authors equally contributed to this work.

[*]Co-corresponding Authors' Emails: Ho-Wook Jun (hwjun@uab.edu) & Dong Woon Kim (visnu528@cnu.ac.kr)

Methods.

Contact Angle Measurement: To assess the surface wettability of the coated silicone implants compared to uncoated silicone, contact angle was measured. To measure the static contact angle, uncoated, 30-layers, and 60-layers bionanomatrix coated silicone (n = 3) were mounted onto a glass slide. Contact angles were measured using the sessile drop method with a water droplet size of 5 μL. Image J software was used to accurately measure the contact angle of the water droplets on the surface.

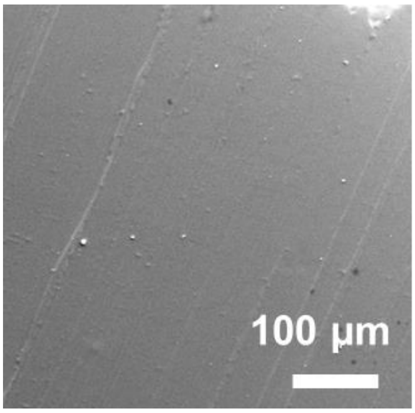

Fig. S1. Scanning electron microscopy (SEM) image of uncoated silicone surface (scale bar: 100 μm).

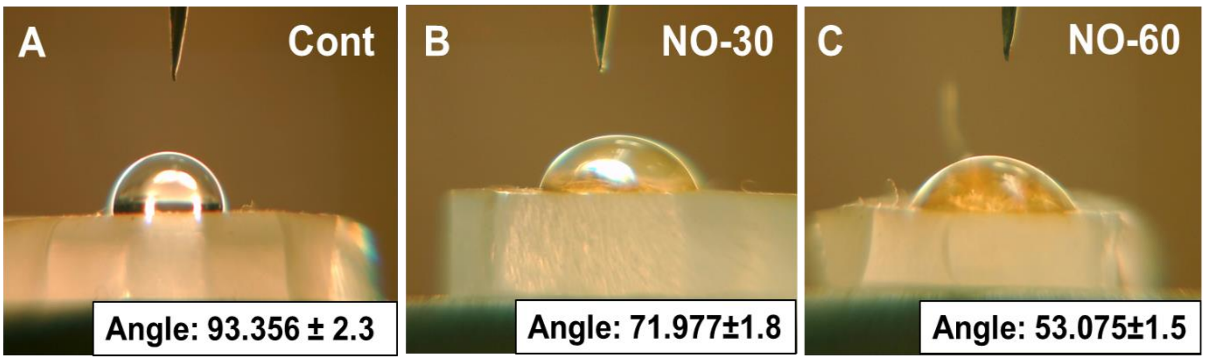

Fig. S2. Contact angle measurement of (a) uncoated (Cont), (b) 30-layer bionanomatrix coated (NO-30), and (c) 60-layer bionanomatrix coated (NO-60) silicone implants.
